# Orai1 is an Entotic Ca^2+^ Channel for Non‐Apoptotic Cell Death, Entosis in Cancer Development

**DOI:** 10.1002/advs.202205913

**Published:** 2023-03-24

**Authors:** Ah Reum Lee, Chan Young Park

**Affiliations:** ^1^ Department of Biological Sciences Ulsan National Institute of Science and Technology Ulsan 44919 Republic of Korea

**Keywords:** calcium signaling, cell‐in‐cell, entosis, Orai1, SEPTIN

## Abstract

Entosis is a non‐apoptotic cell death process that forms characteristic cell‐in‐cell structures in cancers, killing invading cells. Intracellular Ca^2+^ dynamics are essential for cellular processes, including actomyosin contractility, migration, and autophagy. However, the significance of Ca^2+^ and Ca^2+^ channels participating in entosis is unclear. Here, it is shown that intracellular Ca^2+^ signaling regulates entosis via SEPTIN‐Orai1‐Ca^2+^/CaM‐MLCK‐actomyosin axis. Intracellular Ca^2+^ oscillations in entotic cells show spatiotemporal variations during engulfment, mediated by Orai1 Ca^2+^ channels in plasma membranes. SEPTIN controlled polarized distribution of Orai1 for local MLCK activation, resulting in MLC phosphorylation and actomyosin contraction, leads to internalization of invasive cells. Ca^2+^ chelators and SEPTIN, Orai1, and MLCK inhibitors suppress entosis. This study identifies potential targets for treating entosis‐associated tumors, showing that Orai1 is an entotic Ca^2+^ channel that provides essential Ca^2+^ signaling and sheds light on the molecular mechanism underlying entosis that involves SEPTIN filaments, Orai1, and MLCK.

## Graphical abstract

Entosis is a non‐apoptotic cell death process that forms characteristic cell‐in‐cell structures in cancers, killing invading cells. Intracellular Ca^2+^ signaling by IP_3_R coupled Orai1 Ca^2+^ channels regulates entosis via SEPTIN‐Orai1‐Ca^2+^/CaM‐MLCK‐actomyosin axis. This provides insights into a therapeutic target for entotic regulators promoting entosis‐mediated tumor development.

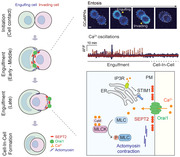

## 1 Introduction

Entosis, a non‐apoptotic cell death process, engulfs homotypic living cells.^[^ ^1^ ^]^ Although it was first observed over 100 years ago, the underlying mechanisms and functional consequences have been studied in recent years. Entosis is triggered by several physiological conditions such as matrix detachment, aberrant mitosis,^[^ ^2^ ^]^ and glucose deprivation.^[^ ^3^ ^]^ These various pathways for entosis reflect the characteristics of cancer cells, such as abnormal proliferation, anchorage‐independence, and metabolic stress, suggesting that intrinsic properties of cancer cells or their microenvironments can cause entosis. It is associated with a wide range of human diseases, such as genome instability,^[^ ^4^, ^5^ ^]^ developmental disorders,^[^ ^6^, ^7^ ^]^ and cancer.^[^ ^8^ ^]^

Regardless of the mechanism by which entotic cells initiate, they form adherens junctions (AJs) via Ca2+/E‐cadherins.^[^ ^9^ ^]^ Following the formation of AJs, they are engulfed through actin polymerization following AJ,^[^ ^10^ ^]^ mechanical ring,^[^ ^11^ ^]^ and actomyosin contraction.^[^ ^1^, ^9^ ^]^ Lastly, they appear in cell‐in‐cell (CIC) structures, resulting in inside cells escaping and dividing; although, they typically die via lysosomal degradation.^[^ ^12^ ^]^

Engulfing (outer, host, winner) and invading (inner, internalizing, engulfed, loser) entotic cells form CIC structures through actin polymerization and myosin contraction. Invading cells have higher stiffness with accumulated actomyosin in the cell cortex, opposite to AJ, and the resulting mechanical tension drives CIC invasion. It is regulated by RhoA, Rho‐associated protein kinase (ROCK), and diaphanous‐related formin 1 (Dia1).^[^ ^13^, ^14^ ^]^ Engulfing cells have lower tension than invading cells and swallow neighboring cells with actin‐dependent engulfment activity. Rac1, a key mediator of the actin cytoskeleton, and KRas, the oncogene regulating Rac1 activity, enhance engulfing by regulating actomyosin contractility.^[^ ^13^ ^]^

Cytosolic Ca^2+^ function as intracellular signaling messengers and are involved in cytoskeleton rearrangement, metastasis, metabolism, cell death, and cancer development.^[^ ^15^, ^16^, ^17^, ^18^ ^]^ An important mechanism for maintaining intracellular Ca^2+^ levels is store‐operated Ca^2+^ entry (SOCE), which is mediated by Orai1 Ca^2+^ channels in plasma membrane (PM)^[^ ^19^, ^20^ ^]^ and stromal interaction molecule (STIM) proteins^[^ ^21^ ^]^ in the endoplasmic reticulum (ER). STIM senses ER Ca^2+^ concentrations and accumulates at ER‐PM junctions following ER Ca^2+^ depletion, activating Orai1 channels in adjacent PM. Ca^2+^ entry through Orai1 channels is associated with several cell signaling processes in most cells.^[^ ^22^, ^23^ ^]^

Orai1‐STIM1 assembly and stability, and architecture of ER‐PM junctions, are determined by specific protein and lipid components including SEPTINs, phosphatidyl 4,5‐bisphosphate(PIP_2_), and proteins scaffolding ER to PM.^[^ ^24^, ^25^ ^]^ SEPTINs, filament forming GTPases, bind to inner PM through specific interactions with PIP_2_ and assemble on membrane domains.^[^ ^26^, ^27^, ^28^ ^]^ They promote stable recruitment of Orai1 by maintaining PIP_2_ organization in PM.^[^ ^29^ ^]^ Their loss results in abnormal Orai1 clustering and reduced SOCE.

Myosin light chain (MLC) phosphorylation is essential for entosis providing contractile force. It is determined by myosin light chain kinase (MLCK), ROCK, and MLC phosphatase (MLCP). MLCK, the best‐studied factor regulating MLC phosphorylation, is Ca^2+^/Calmodulin‐dependent serine/threonine kinase (CaMK) and is activated by CaM in response to an increase in intracellular Ca^2+^ levels. Activated MLCK phosphorylates the regulatory myosin light chains of myosin II (MLC2), specifically at serine‐19, facilitating actin‐activated myosin and promoting myosin‐driven contraction.

Extracellular Ca^2+^ is required for E‐cadherin‐mediated cell junction formation during entosis. However, the role of intracellular Ca^2+^ signaling in entosis, and the mechanism through which it is modulated, remain poorly understood. Entosis‐associated molecular pathways are Ca^2+^‐dependent. Orai1, a major Ca^2+^ route, regulates diverse processes related to entosis. For example, Ca^2+^ influx through Orai1 controls actin organization and dynamics at the immune synapse in T lymphocytes,^[^ ^30^ ^]^ membrane blebs in amoeboid cells,^[^ ^31^ ^]^ and leading‐edge in migrating cells.^[^ ^32^, ^33^ ^]^ Orai1‐dependent signaling enhances contractile force by regulating actomyosin reorganization.^[^ ^22^, ^34^ ^]^ Furthermore, entosis‐associated proteins, including actin, MLC, Ezrin,^[^ ^31^ ^]^ Rac1,^[^ ^33^ ^]^ AMP‐activated protein kinase (AMPK), and vinculin^[^ ^22^ ^]^ are regulated by Orai1 or Orai1 mediating Ca^2+^ signals. We hypothesized that Orai1 is a major route for Ca^2+^ influx during entosis.

Here, we provide evidence that Orai1 is an entotic Ca^2+^ channel, whose membrane localization is tightly controlled by SEPTINs, resulting in temporal entotic Ca^2+^ oscillations that affect MLCK‐actomyosin rearrangement during entosis. We identify the mechanism through Orai1‐mediated Ca^2+^ signaling regulating Ca^2+^‐dependent engulfment during entosis and provide insights into a therapeutic target for entotic regulators promoting entosis‐mediated tumor development.

## 2 Results

### 2.1 Spontaneous Ca^2+^ Oscillations Occur During Entosis

**Figure 1:**
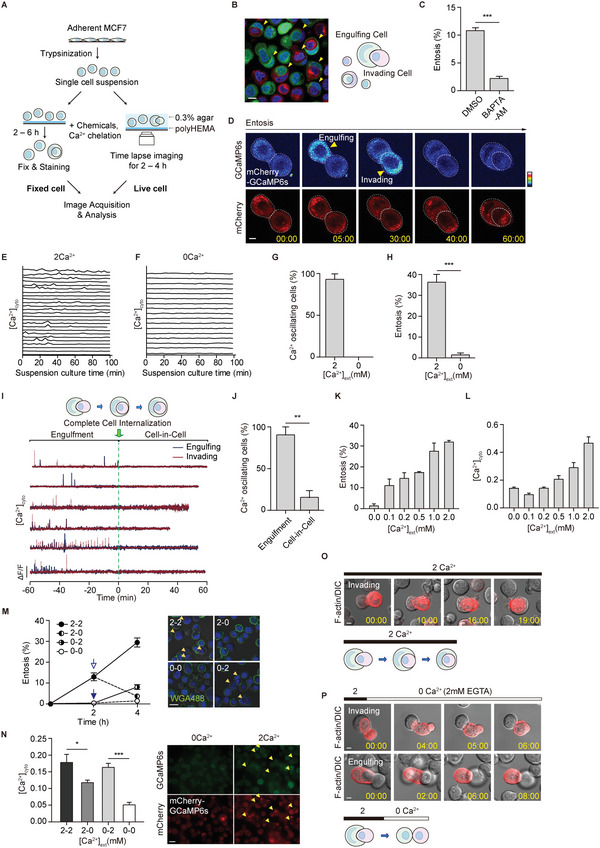
Entotic Ca^2+^ oscillations occur during the engulfment. A) Schematic representation of the method for quantifying and imaging entotic cells. B) Representative images of entotic cells and a diagram of an entotic cell pair: engulfing and invading cells. Yellow arrowheads indicate entotic cells. Scale bar = 10 µm. C) Quantification of internalizing cells in BAPTA‐AM (10 µm, with 100 µm of EGTA) after 6 h of suspension. Data represent mean ± SEM of three independent experiments (*n* > 200 cells). D) Spontaneous Ca^2+^ oscillations in MCF7 cells during entosis. Yellow arrowheads indicate Ca^2+^ signals. Time is presented in min: s. Scale bar = 5 µm. E,F) Graph of normalized GCaMP6s ratio (GCaMP6s/mCherry) in entotic cell pairs exposed to 2 mm extracellular Ca^2+^ (E, from Figure S1A,B, Supporting Information, *n* = 24) and non‐entotic cells exposed to 0 mm extracellular Ca^2+^ (2 mm EGTA) (F, *n* = 17). G) Quantification of Ca^2+^ oscillating cells from (E,F). H) Quantification of internalizing cells cultured in suspension for 4 h in the presence of 2 and 0 mm Ca^2+^. Data represent mean ± SEM of triplicate experiments (*n* > 300 for each cell line). I) Spontaneous Ca^2+^ oscillations in MCF7 cells during entosis. *Y* axis: 4 (A.U.) J) Quantification of Ca^2+^ oscillating cells during engulfment and CIC stages. The timepoint at which a complete CIC structure was made was aligned to 0. (Engulfment: −60 to 0 min, cell‐in‐cell:0–60 min). Data represent mean ± SEM of triplicate experiments (*n* = 7, 6, and 5). K,L) Quantification of internalizing cells depending on extracellular Ca^2+^ concentration (K) and intracellular Ca^2+^ level (GCaMP6s/mCherry) (L). Data represent mean ± SEM of triplicate experiments (*n* = 52 for each condition). M) Quantification of internalizing cells depending on extracellular Ca^2+^ concentration. Ca^2+^ add‐back (0–2) and Ca^2+^ withdrawal (2–0). Data represent mean ± SEM of triplicate experiments (*n* > 200 for each cell line). Scale bar = 20 µm. N) Graph of normalized GCaMP6s ratio (GCaMP6s/mCherry). Data represent mean ± SEM. *n* = 74, 42, 38, and 33 for each group. Scale bar = 20 µm. O,P) Time‐lapse images of entotic cells in the presence of Ca^2+^ (O) and under Ca^2+^ withdrawal (2 → 0 mm and addition of 2 mm EGTA) (P). Cherry‐Lifeact labeled cell morphology. Scale bar = 5 µm. Significance was determined using unpaired two‐tailed *t*‐test. ***p < 0.001; **p < 0.01; *p < 0.05.

Ca^2+^ signaling drives intracellular processes and communicates between cells. Intracellular Ca^2+^ concentration exhibits diverse spatiotemporal dynamics, influencing the versatility of Ca^2+^‐dependent signaling.^[^ ^18^ ^]^ Hence, we investigated whether and how Ca^2+^ signaling, particularly Ca^2+^ channel‐mediated Ca^2+^ dynamics, is required for entosis. To determine whether intracellular Ca^2+^ is necessary for entosis, we first measured the entosis efficiency in MCF7 cells treated with BAPTA‐AM, a cell‐permeable Ca^2+^ chelator. For entosis quantification, adherent MCF7 cells were trypsinized into single cells and cultured in suspension for 2–6 h under the indicated conditions (Ca^2+^ chelation or chemical treatment) (**Figure** **1**A). Cells were fixed and stained for PM and nucleus. Analysis using confocal microscopy demonstrated the complete CIC structures (entotic cells). The percentage of entotic cells was determined through quantifying the number of single cells and CIC structures (Figure 1B). When we treated BAPTA‐AM, the cells showed reduced entosis efficiency compared with cells in the Ca^2+^ medium, confirming that intracellular Ca^2+^ is required for entosis. The cells showed reduced entosis efficiency compared with cells in the Ca^2+^ medium (Figure 1C), confirming that intracellular Ca^2+^ is required for entosis.

To assess the effect of Ca^2+^ signaling on entosis progression, we measured intracellular Ca^2+^ concentrations in “engulfing” and “invading” entotic MCF7 cells expressing mCherry‐GCaMP6s, a genetically encoded Ca^2+^ indicator.^[^ ^35^ ^]^ Time‐lapse microscopy imaging was performed for 100 min at 3–5 min intervals in 0.3% low melting agarose as described^[^ ^1^ ^]^ (Figure 1A,D; Movie S1, Supporting Information). A Ca^2+^‐insensitive fluorophore, mCherry, ensured proper GCaMP6s expression and normalized GCaMP6s intensity. Ca^2+^ oscillations 1.5× above the standard deviation of the threshold of the GCaMP6s/mCherry fluorescence ratio were counted as Ca^2+^ signals. Interestingly, we observed spontaneous Ca^2+^ oscillations in most (90%) entosis proceeding cells in Ca^2+^ dependent manner (Figure 1D–G) as no oscillation occurred when extracellular Ca^2+^ was depleted with 2 mm EGTA (Figure 1F,G) and the efficiency of entosis was reduced in the absence of Ca^2+^ (35% to 1%, Figure 1H). In addition, there were no differences in Ca^2+^ oscillation between “Engulfing” and “Invading” cell pairs (Figure S1, Supporting Information), implying that both cells require intracellular Ca^2+^ signals to undergo entosis. These results suggest that extracellular Ca^2+^ is necessary for initiating entosis and for intracellular Ca^2+^ oscillations during entosis.

### 2.2 Spontaneous Ca^2+^ Oscillations Occur Mainly in the Early to Mid‐Engulfment Stage

We explored entotic Ca^2+^ oscillation by analyzing the efficiency and time kinetics of the initiation of internalization and engulfment. Internalization occurred ≈2 h after matrix detachment (Figure S2A,B, Supporting Information) and was followed by engulfment for 30 to 60 min (Figure S2B,C, Supporting Information). To elucidate temporal Ca^2+^ oscillation patterns during entosis, time points at which entotic cells formed complete CIC structures were set to 0. We monitored changes in Ca^2+^ concentrations in MCF7 cells expressing mCherry‐GCaMP6s 1 h before and after complete cell internalization at 3 s intervals (Figure 1I). Approximately 90% of entotic cells showed spontaneous Ca^2+^ oscillations, with non‐synchronized patterns between engulfing and invading cells, before forming complete CIC structures (Figure 1I,J). Interestingly, we noticed that Ca^2+^ oscillations dramatically disappeared in entotic cells with complete CIC structure. These results indicated that Ca^2+^ oscillations might be temporally controlled during entosis, specifically during engulfment, rather than during most completed CIC structure stages.

### 2.3 Extracellular Ca^2+^ Regulates Intracellular Ca^2+^ Level for Entosis

To determine whether intracellular Ca^2+^ oscillations depend on extracellular Ca^2+^, we measured entosis efficiency and intracellular Ca^2+^ concentrations under various extracellular Ca^2+^ conditions. Both entosis efficiency (Figure 1K) and intracellular Ca^2+^ concentrations (Figure 1L) increased as extracellular Ca^2+^ concentrations increased from 0 to 2 mm, demonstrating that intracellular entotic Ca^2+^ oscillations are linked to extracellular Ca^2+^.

To further confirm the effect of extracellular Ca^2+^ on entosis efficiency and intracellular Ca^2+^ concentrations, we performed “Ca^2+^ add‐back” and “Ca^2+^ withdrawal” in entotic MCF7 cells. We induced entosis in 0 mm extracellular Ca^2+^ with Ca^2+^ chelator EGTA for 2 h of suspension culture and followed by 2 mm Ca^2+^ medium for another 2 h. Entosis efficiency increased from 1% at 2 h to 9% at 4 h (Figure 1M). Intracellular Ca^2+^ concentrations increased approximately threefold after adding Ca^2+^ (Figure 1N). Replacing Ca^2+^ with EGTA (“Ca^2+^ withdrawal”) decreased entosis efficiency from 15% at 2 h to 4% at 4 h (Figure 1M), while efficiency increased from 15% to 30% in Ca^2+^ medium (Figure 1M). We observed similar changes in intracellular Ca^2+^ concentrations with Ca^2+^ withdrawal resulting in an approximately twofold reduction in intracellular Ca^2+^ concentrations (Figure 1N). These results confirmed that extracellular Ca^2+^ influences entosis efficiency by regulating intracellular Ca^2+^ concentrations.

We then visualized how Ca^2+^ regulates the movement of entotic cells using mCherry‐tagged LifeAct. Time‐lapse imaging showed changes in movement and morphology of entotic cells. Half invading entotic cells continued to invade engulfed entotic cells, in the presence of 2 mm extracellular Ca^2+^, resulting in CIC structures (Figure 1O). However, the cells began to emerge soon after inhibiting Ca^2+^ with EGTA (Figure 1P). Interestingly, we found that a pair of almost engulfed entotic cells (completed more than 90%) were not separated into individual cells even in 0 mm extracellular Ca^2+^ with 2 mm EGTA condition (Figure S3, Supporting Information) as proposed.^[^ ^11^ ^]^ It indicated that Ca^2+^ may be necessary for CIC attachment at the initial stage and Ca^2+^ oscillation in the middle stage, but less in the final stage.

Taken together, these results suggested that extracellular Ca^2+^ affects entosis efficiency by regulating oscillating intracellular Ca^2+^ signaling for regulating particular entosis stages.

### 2.4 SOC Channel Blockers Prevent Entosis

**Figure 2:**
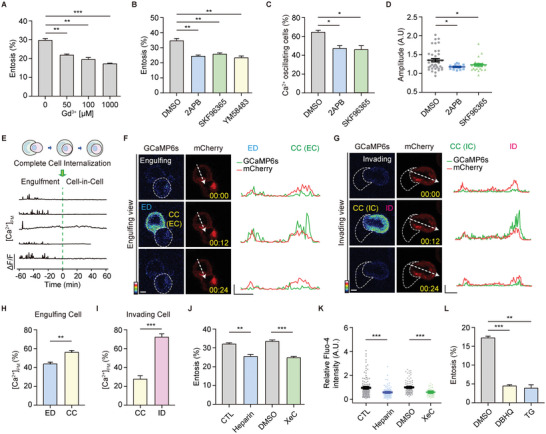
Entotic cells show polarized Ca^2+^ signals during entosis. A) Bar graph showing entosis efficiency after 3 h. Entotic cells were quantified with Gd^3+^ in the dose‐dependent manner. Data represent mean ± SEM of triplicate experiments (*n* > 200 for each). B) Quantification of entotic cells in SOC channel blockers; 2‐APB (50 µm), SKF96365 (10 µm), and YM58483 (10 µm) compared with the control (DMSO). Cell suspensions were cultured with each inhibitor for 4 h. Data represent mean ± SEM of the triplicate experiments (*n* > 300 in each experimental group). C) Quantification of Ca^2+^ oscillating cells in DMSO, 2‐APB, and SKF96365 from (Figure S4, Supporting Information). Higher Ca^2+^ levels, defined by setting the threshold to 1.5× above the standard deviation, were counted as Ca^2+^ signals (*n* = 28, 19, and 24). D) Quantitative comparisons of spontaneous Ca^2+^ signals measured using GCaMP6s (GCaMP6s/mCherry). The compared quantities were peak amplitudes ∆*F*/*F* ratios. Data represent mean ± SEM. E) Graph of normalized GCaMP6s‐CAAX ratio in entotic cells. F,G) Local Ca^2+^ influx was reported using GCaMP6s‐CAAX, the PM Ca^2+^ indicator, in engulfing (F) and invading (G) cells. Line scan profile analysis of mCherry‐GcaMP6s‐CAAX signal. Scale bar = 5 µm. *X* axis: 5 µm, *Y* axis: 500. H,I) Bar graphs of the Ca^2+^ peak amplitudes in cell contact site and distal region of engulfing (*n* = 5) (H) and invading (*n* = 4) cells (I). Data represent mean ± SEM. J) Quantification of entotic cells cultured for 4 h with IP_3_R inhibitor, heparin (400 µg mL^−1^), and Xestospongin C (4 µm). Data represent mean ± SEM of triplicate experiments (*n* > 300 in each experimental group). K) Intracellular Ca^2+^ levels of MCF7 cells in suspension with control (*n* = 124) and heparin (400 µg mL^−1^, *n* = 102), and DMSO (*n* = 93) and Xestospongin C (4 µm, *n* = 59). Fluo‐4 intensity quantification data represent mean ± SEM. L) Quantification of entotic cells cultured for 2.5 h in SERCA inhibitors, DBHQ (25 µm), and TG (1 µm). Data represent mean ± SEM of duplicate experiments (*n* > 200 in each experimental group). Significance was determined using unpaired two‐tailed *t*‐test. ***p < 0.001; **p < 0.01; and *p < 0.05. ED: engulfing cell distal region; CC: cell‐cell contact site; ID: invading cell distal region.

It was surprising to find that no reports have been found regarding Ca^2+^ channels that are associated with entosis. Thus, we explored which Ca^2+^ channels regulate entotic Ca^2+^ signaling for entosis. To find out potential Ca^2+^ channels, suspended MCF7 were treated with gadolinium (Gd^3+^), an inorganic Ca^2+^ channel blocker for 3 h and showed a decreased entosis efficiency (**Figure** **2**A), indicating the involvement of Ca^2+^ channels in entosis. In cancer cells, SOC (Orai) channels are best known for major Ca^2+^ channels. Therefore, MCF7 cells were treated with SOC channel blockers, 2‐APB, SKF96365, and YM58483 for 4 h and showed reduced entosis efficiency from 35% to 20% (Figure 2B), indicating that SOC Ca^2+^ channels might be an unrevealed Ca2+ channel of entosis. We further analyzed the change in Ca^2+^ oscillations in MCF7 cells with the blockers. The number of Ca^2+^ oscillating cells decreased by 45% in the presence of SOC channel blockers compared with DMSO (≈65%, Figure 2C; Figure S4, Supporting Information). The amplitude of intracellular Ca^2+^ transients also decreased (Figure 2D). These results provide the first evidence that SOC channels are entotic Ca^2+^ channels that can induce Ca^2+^ oscillations.

### 2.5 Entotic Cells Show Polarized Membrane Ca^2+^ Signals

Ca^2+^ concentrations of entotic Ca^2+^ oscillating cells were enriched in the PM of both invading as well as engulfing cells (Figure 1D); thus, we analyzed spatially localized Ca^2+^ signaling during entosis using mCherry‐GCaMP6s‐CAAX, a PM‐localized Ca^2+^ indicator. Examining the localization and Ca^2+^ responsiveness of mCherry‐GCaMP6s‐CAAX in MCF7 cells showed restricted local Ca^2+^ influx without Ca^2+^ release from depleted stores (Figure S5, Supporting Information), indicating that GCaMP6s‐CAAX can measure Ca^2+^ changes near PM. To investigate entotic membrane Ca^2^ signaling in detail, we performed time‐lapse imaging. In MCF7 cells expressing mCherry‐GCaMP6s‐CAAX, the spontaneous Ca^2+^ oscillations were PM‐enriched and spatially localized in both engulfing and invading entotic cells (Figure 2E–G), which is similar to the results of GCaMP6s, a cytosolic Ca^2+^ indicator, during engulfment (Figure 1I).

We further analyzed the concentration of membrane Ca^2+^ of engulfing and invading cells. Interestingly, not only a change in the local Ca^2+^ distribution but also a differential local distribution was observed between invading and engulfing cells during engulfment. In engulfing cells, local Ca^2+^ concentrations were higher at CC (cell–cell contact site) than at the distal membranes (engulfing cell distal region, ED) (Figure 2F,H; Movie S2, Supporting Information). However, invading cells had a higher level of Ca^2+^ at the cell cortex of the distal membrane (invading cell distal region, ID) and lower local Ca^2+^ concentrations at the CC (Figure 2G,I; Movie S3, Supporting Information). These results provide the first evidence that differential Ca^2+^ signaling in entotic cell pairs is required for proper cellular signaling and organization during entosis.

### 2.6 IP_3_ Related Store Depletion Could Induce Entotic Ca^2+^ Signaling

To check how Ca^2+^ oscillations are induced and dependent on store depletion during entosis, we investigated whether intra‐ER Ca^2+^ signaling through the inositol 1,4,5‐trisphosphate receptor (IP_3_R) or sarco/endoplasmic reticulum Ca^2+^‐ATPase (SERCA) may affect the Ca^2+^ influx and entosis efficiency. First, we treated MCF7 cells with the IP_3_R inhibitor, heparin (400 µg mL^−1^), and Xestospongin C (4 µm), and measured intracellular Ca^2+^ concentration and entosis efficiency. The cells with the IP_3_R inhibitor showed a reduced entosis efficiency (Figure 2J), as well as a decrease in the intracellular Ca^2+^ concentration (Figure 2K), indicating that IP_3_R coupled store depletion followed by SOCE activation could play a role in entosis. In addition, we depleted ER Ca^2+^ by treating the cells with DBHQ (Di‐tert‐butylhydroquinone) or TG (Thapsigargin), two SERCA inhibitors. Our results showed that DBHQ (25 µm) or TG (1 µm) significantly reduce the efficiency of entosis (Figure 2L), indicating that SERCA‐mediated ER Ca^2+^ maintenance is crucial for entosis.

Therefore, these results suggest that intra‐ER Ca^2+^ signaling through the IP_3_R is critical for SOCE activation which leads to generating Ca^2+^ oscillations in entosis.

### 2.7 Orai1 is the Entotic Ca^2+^ Channel

**Figure 3:**
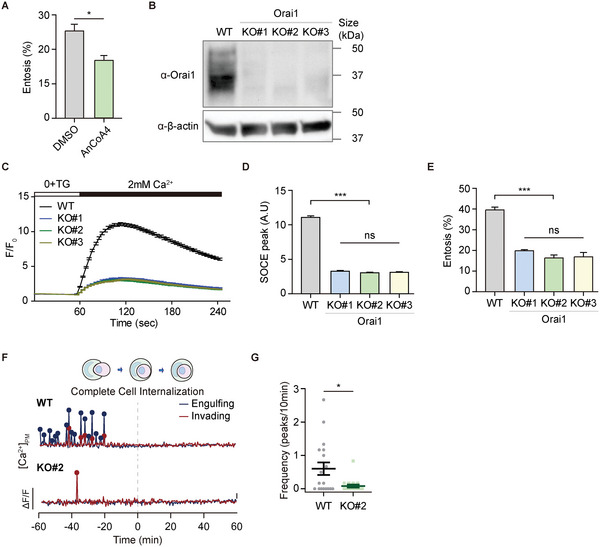
Orai1 is an unrecognized entotic Ca^2+^ channel. A) The effect of AnCoA4 on entosis. The cells were pretreated with AnCoA4 (10 µm) and DMSO for 24 h and quantified entotic cells after 3 h suspension. Data represent mean ± SEM of triplicate experiments (*n* > 200 for each). Significance was determined using unpaired two‐tailed *t*‐test. *p < 0.05. B) Western blot analysis of Orai1 WT and KO MCF7 cells. *β*‐actin was included as endogenous control. C) TG‐induced Ca^2+^ influx in Orai1 KO cell lines monitored by Fluo‐4 (*F*/*F*_0_). D) Comparison between the SOCE peaks from (C). Data represent mean ± SEM (*n* = 165, 67, 136, and 148 cells from each cell line). Statistical analysis was performed using one‐way ANOVA followed by Dunnett's test. ***p < 0.001. ns: not significant. E) Quantification of entotic cells in Orai1 WT and KO MCF7 cells. Data represent mean ± SEM of the triplicate experiments (*n* > 300 in each experimental group). Statistical analysis was performed using one‐way ANOVA followed by Dunnett's test. ***p < 0.001. ns: not significant. F) Local Ca^2+^ influx reported using mCherry‐GCaMP6s‐CAAX in Orai1 WT and KO MCF7 cells. G) Quantification of Ca^2+^ signals in Orai1 WT and KO entotic cells (*n* = 18 for each). Three independent experiments were quantified. Significance was determined using unpaired two‐tailed *t*‐test. *p < 0.05.

We hypothesized that this polarized membrane Ca^2+^ might be induced by Orai1 Ca^2+^ channels, the best‐known SOC channel because it regulates lots of common signaling pathways in cancer cells that are also essential for entosis, including actin polymerization, myosin contraction, and membrane blebbing. To test this, we treated MCF7 cells with 10 µM AnCoA4 (**Figure** **3**A), an Orai1‐specific inhibitor that binds directly to the C‐terminus region of Orai1 and blocks Ca^2+^ influx.^[^ ^36^ ^]^ It is not surprising that we observed a decrease in entosis efficiency, indicating that the Orai1 channel may be an entotic Ca^2+^ channel.

We used CRISPR‐Cas9‐generated Orai1 knockout (KO) MCF7 cells to explore the function of Orai1 Ca^2+^ channels during entosis (Figure S6A,B, Supporting Information). We confirmed the ablation of Orai1 protein expression in three representative Orai1 KO cell lines with different indel (deletion/insertion) mutations using immunoblotting (Figure 3B) and immunocytochemistry (Figure S6C, Supporting Information). We confirmed that the loss of Orai1 resulted in significantly reduced SOCE when ER Ca^2+^ stores were depleted (Figure 3C,D). There were no significant differences in other SOCE components (STIM1, STIM2, Orai2, Orai3) between wildtype (WT) and KO cells (Figure S6D–G, Supporting Information).

We next found that Orai1 deletion decreased entosis efficiency from 40% to 15% (Figure 3E), indicating that Orai1 Ca^2+^ channels positively regulate entosis. To investigate whether Orai1 Ca^2+^ channels induce local Ca^2+^ influx in entotic cells during the engulfment stages of entosis, we expressed mCherry‐GCaMP6s‐CAAX in Orai1 WT and KO cells, acquired time‐lapse images at 30 s intervals over 4 h, and aligned the end of engulfment to time 0. WT cells showed PM‐localized Ca^2+^ oscillations before entotic cells formed CIC structures (engulfment stage: ≈ −60 to 0 min) (Figure 3F; Movie S4, Supporting Information). Surprisingly, few oscillating cells were detected and the frequency of Ca^2+^ oscillations was significantly lower in Orai1 KO cells (Figure 3F,G; Movie S5, Supporting Information), suggesting that Orai1 is a bona fide entotic Ca^2+^ channel that provides local Ca^2+^ oscillations in entotic cells for the engulfment.

### 2.8 Orai1 Shows the Polarized Distribution in Invading Cells

**Figure 4:**
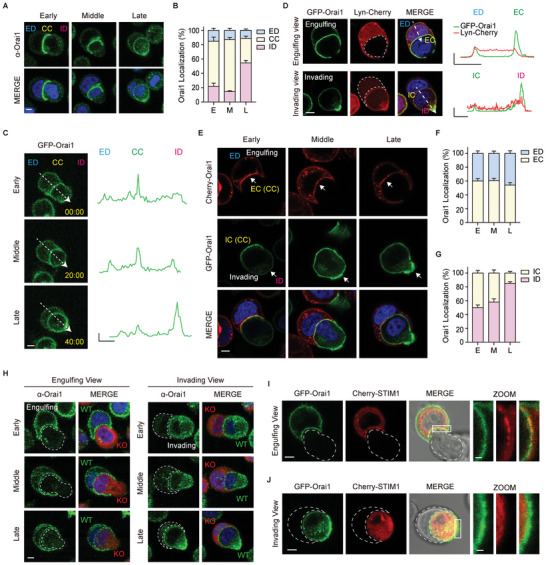
Orai1 shows the polarized distribution during entosis. A) Immuno‐fluorescent images of suspended cells taken after 2.5 h of culturing show endogenous Orai1 (green). B) Bar graphs showing the distribution of Orai1 at different engulfment stages. Early‐stage (E, <1/3 internalization, *n* = 10); middle‐stage (M, 1/3–2/3 internalization, *n* = 24); late‐stage (L, >2/3 internalization, *n* = 35). C) Time‐lapse fluorescence images of GFP‐Orai1 stably expressed MCF7 cells during entosis. Times are indicated as h: min: s. Line graphs show GFP pixel intensities for the indicated line scans. *X* axis: 5 µm, *Y* axis: 5000 (A.U.). D) Fluorescence images of GFP‐Orai1 and Lyn‐Cherry (PM marker) expressing MCF7 cells. Line scan analysis of relative GFP‐Orai1 (green) and Lyn‐Cherry (red) signals along the white arrow in the merge image. *X* axis: 5 µm, *Y* axis: 1 (A.U.). E) Fluorescence images of Cherry‐Orai1 (engulfing cell)‐ and GFP‐Orai1 (invading cell)‐expressing MCF7 cells cultured for 2.5 h in suspension. F,G) Bar graphs showing Orai1 distribution in engulfing cells (F) and invading cells (G). H) Immunofluorescence images of endogenous Orai1 (green) in WT and Orai1 KO (red) cells show Orai1 distribution in engulfing and invading cells. I,J) Fluorescence images of GFP‐Orai1‐ and Cherry‐STIM1‐ expressing MCF7 cells cultured for 2.5 h in suspension. Engulfing (I) and Invading (J) cells. Cropped image scale bar = 1 µm. Scale bar = 5 µm. ED: engulfing cell distal region; CC: cell–cell contact site; ID: invading cell distal region. EC: engulfing cell contact site. IC: invading cell contact region.

To determine whether Orai1 Ca^2+^ channels affect Ca^2+^ concentrations in entotic cells, we analyzed the localization of endogenous Orai1 at early, middle, and late engulfment stages, based on the proportion of engulfment membrane (**Figure** **4**A). Similar to the observation that entotic cells have differential Ca^2+^ concentrations with mCherry‐GCaMP6s‐CAAX (Figure 2F–I), we observed preferential localization of Orai1 during engulfment. Approximately 60% of Orai1 accumulated at CC in the early and middle engulfment stages and moved to the ID in the late stage (Figure 4B). We tracked the localization of Orai1 in cells expressing GFP‐Orai1 using time‐lapse imaging (Figure 4C; Movie S6, Supporting Information). Although distinct differential localization of endogenous Orai1 or GFP‐Orai1 was not observed in engulfing cells, GFP‐Orai1 was enriched in CC during the early engulfment stage and translocated predominantly to the ID following engulfment. Moreover, the polarized localization of Orai1 was confirmed using cells labeled with cytoplasmic membrane dyes (CellBrite Red, Figure S7A, Supporting Information) and cytosol dyes (CellTracker Red, Figure S7B, Supporting Information).

With single fluorescence imaging, it is difficult to determine the differential localization of Orai1 in entotic cell pairs; although, we observed the preferential localization of Orai1 in ID. Therefore, we expressed GFP‐Orai1, respectively in engulfing cell or invading cell and additionally co‐expressed Lyn‐Cherry, PM marker protein, demonstrating the preferential localization of Orai1 during entosis (Figure 4D). Furthermore, we mixed cells expressing mCherry‐Orai1 or GFP‐Orai1 and visualized the localization of Orai1 in entotic cells, having different pairs of fluorescent‐tagged‐Orai1s during engulfment (Figure 4E). Two‐color label assay confirmed that Orai1 was translocated from CC (IC) to ID in invading cells (Figure 4E,G), but engulfing cells appeared preferentially at the CC during entire engulfment stage (Figure 4E,F). The polarized patterns were further confirmed using endogenous Orai1 immunofluorescence imaging (Figure 4H).

Orai1 channels are activated through direct interaction with the ER Ca^2+^ sensor, STIM. Co‐expressing GFP‐Orai1 and Cherry‐STIM1 in MCF7 cells showed that Orai1 and STIM1 are co‐localized during entosis (Figure 4I,J), suggesting that STIM1 activates Orai1, resulting in entotic Ca^2+^ oscillation and cellular responses during engulfment.

Taken together, Orai1 translocation and the spatial entotic Ca^2+^ oscillation are well correlated, suggesting that Orai1, as an entotic Ca^2+^ channel, may regulate spatiotemporal Ca^2+^ signaling in entotic cell pairs during entosis.

### 2.9 SEPT2 Organizes the Distribution of Orai1 in Invading Cells

SEPTINs have been known to modulate local STIM‐ORAI Ca^2+^ signaling by modulating Orai1 concentrations at the ER‐PM junction,^[^ ^29^, ^37^, ^38^ ^]^ which led us to investigate whether SEPTINs regulate Orai1 distribution in entotic cells during entosis.

**Figure 5:**
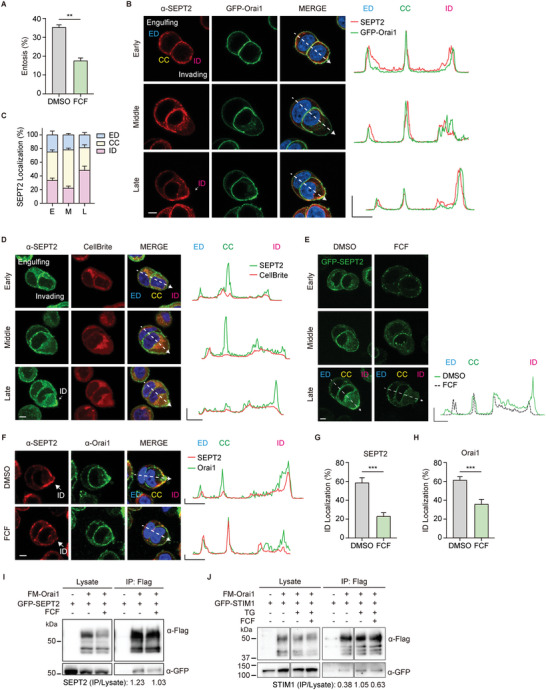
SEPT2 organizes the polarized distribution of Orai1 in invading cells. A) Quantification of entotic cells cultured for 4 h with DMSO and the SEPTIN inhibitor, FCF (50 µm). Data represent mean ± SEM of triplicate experiments (*n* > 300 in each experimental group). B) Immunofluorescence images show endogenous SEPT2 (red) and GFP‐Orai1 (green) in MCF7 cells cultured for 2.5 h in suspension. The arrow indicates enriched SEPT2 at ID. Line graphs show relative SEPT2 (red) and GFP‐Orai1 (green) intensities for the indicated line scans. *X* axis: 5 µm, *Y* axis: 1 (A.U.). C) Bar graph showing the distribution of SEPT2 at different engulfment stages. Early‐stage (E, *n* = 7); middle‐stage (M, *n* = 9); and late‐stage (L, *n* = 13). D) Immuno‐fluorescent images of suspended cells taken after 2.5 h of culturing show endogenous SEPT2 (green) and CellBrite (red, PM marker). Line graphs show relative SEPT2 (green) and PM marker (red) intensities for the indicated line scans. *X* axis: 5 µm, *Y* axis: 1 (A.U.). E) Fluorescence images of GFP‐SEPT2‐expressing MCF7 cells cultured for 2.5 h in DMSO and FCF (50 µm). Line graphs show the distribution of GFP‐SEPT2 in DMSO (green) and FCF (black dotted). F) Immunofluorescence images showing endogenous SEPT2 (red) and endogenous Orai1 (green) in MCF7 cells cultured for 2.5 h in DMSO or FCF (50 µm). The arrow indicates enriched SEPT2 at ID. Line graphs show the distribution of SEPT2 (red) and Orai1 (green). *X* axis: 5 µm, *Y* axis: 5000 (A.U.). G,H) Bar graphs showing the distribution of SEPT2 (G, *n* = 10) and Orai1 (H, *n* = 7) at ID in late engulfment stage (from Figure S10, Supporting Information). I) Immunoblots of whole‐cell lysates (left) or IP (right) from cell co‐expressing Flag‐Myc‐Orai1 and GFP‐SEPT2 in HEK293T cells with or without 50 µm of FCF. J) Immunoblots of whole‐cell lysates (left) or IP (right) from cell co‐expressing Flag‐Myc‐Orai1 and GFP‐STIM1 in HEK293T cells with or without 50 µm of FCF and TG (1 µm). Scale bar = 5 µm. Significance was determined using unpaired two‐tailed *t*‐test. ***p < 0.001; **p < 0.01. ED: engulfing cell distal region; CC: cell–cell contact site; ID: invading cell distal region.

First, we explored the role of SEPTINs during entosis using the SEPTIN inhibitor, forchlorfenuron (FCF; 50 µm), which alters the assembly and disassembly of SEPTIN networks.^[^ ^39^ ^]^ Surprisingly, we found that entosis efficiency in MCF7 cells treated with FCF for 4 h reduced from approximately 35% to 15% (**Figure** **5**A). In addition, we confirmed the unrecognized function of SEPTINs by adding the inhibitor in the middle of entosis. Efficiency decreased to 25% compared with the control (40%) 2 h after FCF was added (Figure S8A, Supporting Information). We also found that FCF also delayed the early‐stage onset of entosis (Figure S8B, Supporting Information). Hence, these results suggest that SEPTINs may play a role in entosis by modulating the preferential localization of Orai1 and local Ca^2+^ oscillations.

Next, we checked whether SEPT2, a core component of SEPTIN filaments,^[^ ^26^, ^40^, ^41^ ^]^ might translocate in entotic cells during engulfment. Surprisingly, we found that SEPT2 is also mainly accumulated at CC during the early and middle and translocated to ID at the late stage of engulfment (Figure 5B,C). The subcellular location and translocation patterns of SEPT2 are similar to those of Orai1 and local Ca^2+^ signaling at different engulfment stages (Figure 5B,C; Figure S9A,B, Supporting Information). Moreover, the polarized localization of SEPT2 was confirmed using cells labeled with cytoplasmic membrane dyes (CellBrite Red, Figure 5D) and cytosol dyes (CellTracker Red, Figure S9C, Supporting Information).

We further confirmed that differential distributions of GFP‐SEPT2 filament structures beneath PM were in MCF7 cells treated with FCF, showing an abnormal GFP‐SEPT2 pattern at CC and ID (Figure 5E). Thus, altered SEPTIN dynamics reduced entosis under FCF conditions (Figure S10A–D, Supporting Information) with abnormal SEPT2 distribution, showing reduced SEPT2 translocation from CC to ID (Figure 5F,G). Thus, SEPT2 might regulate the preferential localization of Orai1 in entotic cells during engulfment, resulting in spatially controlled Ca^2+^ oscillation. Hence, we investigated whether FCF‐treated SEPTIN altered the local distribution of Orai1 during entosis. Orai1 and SEPT2 were co‐localized in MCF7 cells regardless of FCF treatment, indicating that SEPT2 is a strong Orai1 modulating protein and determines the location of Orai1 in entotic cells. Further, FCF reduced the accumulation of Orai1 at ID in the late stage (Figure 5F,H; Figure S10A,B,E,F, Supporting Information).

To determine whether SEPTINs bind to Orai1 and affect the Orai1‐STIM1 complex, we expressed Flag‐Myc‐Orai1 and GFP‐SEPT2 or GFP‐STIM1, and immunoprecipitated them with Flag‐Myc‐Orai1. FCF reduced the interaction between Orai1 and SEPT2 (Figure 5I). GFP‐STIM1 intensity increases through TG treatment (from 0.38 to 1.05) and decreases through FCF treatment (from 1.05 to 0.63), suggesting that STIM1 and Orai1 interaction is store‐dependent and regulated by SEPTINs (Figure 5J). These data indicated that SEPT2 regulates entotic Ca^2+^ signaling via Orai1‐SEPT2 and SOCE complex for the local distribution of Orai1.

These results provide the first evidence that SEPTINs are involved in entosis, stabilizing STIM1‐Orai1 complexes, critical for entotic Ca^2+^ signaling around cell–cell contact and distal membranes of invading cells.

### 2.10 Local Entotic Ca^2+^ Signaling of Orai1 Induces the Local Phosphorylation of MLC

Finally, we explored the molecular mechanism by which the entotic Orai1 Ca^2+^ signaling affects the entotic machinery or target signaling molecules involved in the engulfment processes. We showed that entotic Ca^2+^ signaling is necessary for the morphological changes in cells which are regulated by actin–myosin mediated cytoskeleton rearrangement during engulfment (Figure 1O,P). Myosin activity of actomyosin is regulated by reversible phosphorylation of conserved amino acids, specifically at serine‐19 in myosin light chain (MLC), and determined by the balance between activities of several kinases like MLCK, ROCK, and myosin phosphatase. We hypothesized that local Orai1 Ca^2+^ influx might activate Ca^2+^/CaM/MLCK signaling pathway, resulting in MLC phosphorylation (pMLC) for actomyosin contractility and changes in entotic cell morphology.

**Figure 6:**
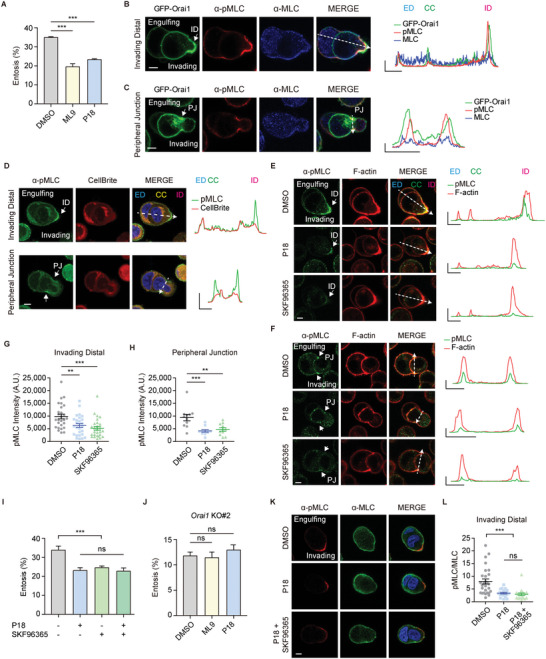
Orai1 regulates Ca^2+^/CaM/MLCK mediated phosphorylation of MLC. A) Quantification of entotic cells cultured for 4 h in DMSO and MLCK inhibitors, ML‐9 (10 µm), and P18 (10 µm). Data represent mean ± SEM of triplicate experiments (*n* > 300 in each experimental group). Significance was determined using unpaired two‐tailed *t*‐test. ***p < 0.001. B,C) Immuno‐fluorescence images of suspended cells after 2.5 h of culturing show endogenous pMLC (red) and MLC (blue) in GFP‐Orai1‐expressing MCF7 cells. pMLC2 is enriched at ID (B) and PJ (C) compared with total MLC. Line graphs show pMLC, MLC, and GFP intensities for the indicated line scans. *X* axis: 5 µm, *Y* axis: 5000. D) Immuno‐fluorescent images of suspended cells taken after 2.5 h of culturing show endogenous pMLC (green) and CellBrite (red, PM marker). Line graphs show relative pMLC (green) and PM marker (red) intensities for the indicated line scans. *X* axis: 5 µm, *Y* axis: 1 (A.U.). E,F) Immunofluorescence images of endogenous pMLC (green) and F‐actin (phalloidin, red) cultured with DMSO, P18, or SKF96365 at ID (E) and PJ (F). Line graphs show pMLC and F‐actin intensities for the indicated line scans. *X* axis: 5 µm, *Y* axis: 20 000. G,H) Quantification of pMLC2 intensity at ID (G, *n* = 27 in each group) and PJ (H, *n* = 11 in each group). Significance was determined using unpaired two‐tailed *t*‐test. ***p < 0.001; **p < 0.01. I) Quantification of entotic cells cultured for 4 h in MLCK inhibitors, P18 (10 µm) and SOC channel inhibitor, SKF‐96365 (10 µm). Data represent mean ± SEM of triplicate experiments (*n* > 300 in each experimental group). Statistical analysis was performed using one‐way ANOVA followed by Dunnett's test. ***p < 0.001. ns: not significant. J) Quantification of entotic cells in Orai1 KO MCF7 cell with MLCK inhibitors, ML‐9 (10 µm) and P18 (10 µm). Significance was determined using unpaired two‐tailed *t*‐test. ns: not significant. Data represent mean ± SEM of triplicate experiments (*n* > 300 in each experimental group). K) Immuno‐fluorescence images of suspended cells after 2.5 h of culturing show endogenous pMLC (red) and MLC (green) in MCF7 cells. L) Quantification of pMLC/MLC intensity ratio at ID (*n* = 28, 20, 19). Statistical analysis was performed using one‐way ANOVA followed by Dunnett's test. ***p < 0.001. ns: not significant. Scale bar = 5 µm. ED: engulfing cell distal region; CC: cell–cell contact site; ID: invading cell distal region; PJ: peripheral junction.

First, we evaluated the influence of MLCK on entosis using two MLCK inhibitors, ML‐9 (10 µm) and peptide‐18 (P18, 10 µm). ML‐9 is a classic MLCK inhibitor and P18 is a specific MLCK inhibitor that mimics the inhibitory domain of MLCK by interacting with the catalytic domain of MLCK. MCF7 cells with inhibitors showed reduced entosis efficiency, demonstrating that MLCK activity and phosphorylation of its downstream protein (MLC) might affect entosis (**Figure** **6**A).

We also examined the local distribution of pMLC as an indicator of active actomyosin bundles as well as Orai1 entotic Ca^2+^ mediators during engulfment. Actomyosin, which is asymmetrically enriched and highly active at the ID, drives cell internalizations by providing contractile force. In addition, actomyosin at the peripheral junction (PJ) of engulfing and invading cells maintains the stability of the AJ complex, which occasionally forms a ring‐like structure with F‐actin. Besides, pMLC was colocalized with Orai1 during engulfment and was enriched in ID (Figure 6B; Figure S11A, Supporting Information) and PJ (Figure 6C; Figure S11B, Supporting Information). Moreover, we confirmed the local MLC phosphorylation using cells labeled with cytoplasmic membrane dyes (CellBrite Red, Figure 6D) and cytosol dyes (CellTracker Red, Figure S11C, Supporting Information). These results implicated the role of Orai1 as an entotic Ca^2+^ mediator for activating actomyosin through local MLC phosphorylation.

We also examined whether Ca^2+^/CaM/MLCK might affect pMLC in Orai1‐enriched locations within entotic cell pairs using MLCK and SOC channel (Orai1) inhibitors. MLCK inhibitor (P18) or SOC channel blockers (SKF96365) decreased pMLC intensity in ID (Figure 6E,G; Figure S11D–G, Supporting Information) and PJ (Figure 6F,H; Figure S11E,F,H, Supporting Information), indicating that suppressing Orai1/Ca^2+^ and MLCK reduces MLCK activity and pMLC in entotic Ca^2+^‐active regions.

To confirm whether SOCE induced entosis through MLCK, we conducted a double inhibition experiment with SOC channel (SKF96365) and MLCK (P18) inhibitors. We compared the efficiency of entosis between cells treated with both inhibitors and against each other. The effects of MLCK and SOC channel inhibitor on the reduction of entosis were not significantly different. In addition, reduced entosis efficiency was not further decreased when both inhibitors were used (Figure 6I). In addition, we checked the effect of MLCK inhibitors on entosis using Orai1 KO cells. The entosis efficiency was not further decreased when two MLCK inhibitors were used in Orai1 KO cells (Figure 6J). Furthermore, we examined the MLC phosphorylation in the double inhibition (Figure 6K,L). MLCK inhibition alone was not significantly different from MLCK and SOC channel double inhibition. Therefore, these results suggest that SOC channel induces entosis through the activation of MLCK.

These findings suggest that Orai1 channel activates CaM/MLCK and myosin (pMLC) in specific cell regions, facilitating engulfment between entotic cells.

## 3 Discussion

In our study, we demonstrated that Orai1 is a critical Ca^2+^ channel that induces Ca^2+^ oscillations during entosis. Orai1 channel is the main pore subunit, but it has two mammalian homologs, Orai2 and Orai3. In addition, Orai1 channel functions as the homomeric or heteromeric channel with other Orai members. Particularly, Orai3 has emerged as a potential fine‐tuner for Ca^2+^ signaling in a variety of cancer cells, including breast cancer cells MCF7.^[^ ^42^ ^]^ Furthermore, several TRP channels were found to have a corporative function with Orai1 in cancer physiology;^[^ ^43^, ^44^ ^]^ however, their function during entosis remains unknown. Ca^2+^ signaling mediated by Orai1 is also modulated by several STIM and Orai binding proteins, especially calcium‐dependent proteins such asCaM,^[^ ^45^ ^]^ SARAF,^[^ ^46^ ^]^ EF‐hand domain family member B (EFHB),^[^ ^47^ ^]^ and Cortactin.^[^ ^33^, ^48^ ^]^ It is, therefore, necessary to investigate the role of Orai1 interacting proteins in entosis in the future.

SOCE activation is dependent on intra‐ER Ca^2+^ signaling through IP_3_R, which leads to Ca^2+^ oscillations during entosis. Entosis is induced via matrix detachment and formation of AJ, which may activate Orai1‐mediated Ca^2+^ signaling. It has been reported that E‐cadherin ligation stabilizes and activates epidermal growth factor receptor (EGFR) signaling^[^ ^49^, ^50^, ^51^ ^]^ and activation of EGFR may result in the activation of phospholipase C gamma and ER Ca^2+^ depletion, resulting in Orai1 mediated Ca^2+^ influx.^[^ ^52^ ^]^ However, several lines of evidence demonstrate that, despite the small conductance of Orai Ca^2+^ channels and high Ca^2+^ selectivity, all Orai isoforms are not non‐redundant for the diversity of Ca^2+^ signaling/oscillations^[^ ^53^ ^]^ and all SOCE components are independently involved in the generation of Ca^2+^ oscillations. Through activation of physical receptors, the diversity of Ca^2+^ oscillations can be expanded, including activation of Src for cancer invasion and fine‐tuning transcriptional activation of the Ca^2+^‐dependent transcription factor Nuclear Factor of Activated T‐cell (NFAT).^[^ ^54^, ^55^ ^]^ Ca^2+^ oscillations are influenced by positive and negative feedback effects on the Ca^2+^ release system, which result in fluctuations in IP_3_ levels or changes in Ca^2+^ channel activity in intracellular stores.^[^ ^56^, ^57^, ^58^ ^]^ Moreover, IP_3_ stimulates the IP_3_R and triggers Ca^2+^ release from the ER, leading to Ca^2+^ influx mediated by Orai1, together with our observations that IP_3_R inhibitor and SERCA inhibitors reduce the entosis efficiency and Ca^2+^ level during entosis. Therefore, it is reasonable to conclude that the Ca^2+^ spikes/oscillations, we observed in entosis, would be IP_3_‐dependent Ca^2+^ oscillations (which last tens to hundreds of seconds), which are associated with the functional coupling between ER Ca^2+^ channels (IP_3_Rs) and PM Orai Ca^2+^ channels.

During CIC formation, Orai1‐mediated Ca^2+^ oscillations occur primarily during the early to the mid‐engulfment stage. It is a question inherent in our results that few Ca^2+^ oscillations were observed in entotic cells following the formation of complete CIC structures (Figure 1I,J). Furthermore, we observed that invading cells displayed Ca^2+^ oscillations during the escape process from CIC structures, implying that Ca^2+^ signaling is also necessary for escape, one of the fates of invading cells. Considering that morphological changes in escaping invading cells require cytoskeletal rearrangement, which requires Ca^2+^ signals, validation of Ca^2+^ signals in CIC structures would be of interest in determining the fate of entotic cells.

Orai1 KO cells showed a reduced entosis with a dramatic decrease in Ca^2+^ oscillations (Figure 3E–G), implying that Orai1‐mediated Ca^2+^ oscillations might be critical but not absolutely essential for entosis. We cannot exclude the possibility of the existence of different types/patterns of Ca^2+^ oscillations or undetectable levels of Ca^2+^ oscillations of varying sizes, intensities, shapes, frequencies, and local distributions, caused by other unrecognized Ca^2+^ pumps, channels, or Orai isoforms (Orai2 and Orai3) present in many types of cells, including MCF7. MCF7 cells express not only Orai1 but also other Ca^2+^ channels (such as Orai2, Orai3, and TRPC channels) which can be involved in Ca^2+^ signaling during the entosis; however, there are no studies about Ca^2+^ channel mediated signalings and whether these Ca^2+^ signalings may or may not be detected levels of oscillations. Thus, further study is needed to expand our understanding on how diverse Ca^2+^ signalings are involved in entosis.

It is important to note that both extracellular and intracellular Ca^2+^ ions and signalings are absolutely essential, particularly during the early stages of engulfment. First, in the absence of extracellular Ca^2+^, Ca^2+^ oscillations did not appear, and entosis occurred very rarely in WT MCF7 cells (less than 1%, Figure 1F–H). Second, when extracellular and intracellular Ca^2+^ were removed from entotic cells which were undergoing entosis, entotic cells ceased to undergo entosis and returned to single cells (Figure 1O,P). Moreover, the Ca^2+^ ions and oscillations are not critical for the late state, which is determined by more than 2/3 of the engulfment state, because entotic cells are still able to form CIC structures even when the Ca^2+^ is withdrawn (Figure S3, Supporting Information).^[^ ^11^ ^]^ Thus, entosis can be triggered by a variety of signaling events, including physical cell‐cell contact by cadherins and Ca^2+^ signaling by Ca^2+^‐handling proteins. Furthermore, during various stages of entosis, individual entotic cells employ a variety of but unknown signaling events; in addition, how Orai1 and other channels cooperate in the progress of entosis will require further investigation.

## 4 Experimental Section

### Cell Lines and Cell Culturing

MCF7 and HEK293T cells were cultured in Dulbecco's modified eagle medium (DMEM) supplemented with 10% fetal bovine serum (FBS) at 37 °C and 5% CO_2_. For transient transfection, the cells were transfected at 70% confluency with 0.2–1 µg DNA using Lipofectamine 3000 (Invitrogen) or jetPRIME (PolyPlus) according to the manufacturer's instructions.

### Cell Internalization Assays and Entosis Quantification

MCF7 cells were cultured for at least three days before the experiment. Monolayer cells were trypsinized to a single‐cell suspension and washed briefly with phosphate buffered saline (PBS). Suspended cells (1–2 × 10^5^ cells per mL) were cultured in the indicated conditions (Ca^2+^ chelation, chemical treatment) for 2–6 h on polyHEMA (Sigma‐Aldrich, 192066) coated plates.^[^ ^1^ ^]^ Cells were pelleted at the indicated times, washed in PBS, and fixed in 4% PFA for 10 min at 25 °C. Fixed samples were washed in PBS and stained with Alexa FluorTM 594 Phalloidin (Invitrogen, A12381), WGA‐488 (Invitrogen, W11261), and Hoechst 33342 (Invitrogen, H3570) for 30 min at 25 °C. To confirm that MCF7 cells were completely internalized by entosis, the cells were examined under a laser‐scanning confocal microscope LSM780 (NLO; Zeiss). Prior to heparin treatment, cells were permeabilized by saponin (10 µg mL^−1^).

The percentage of entotic cells was determined by quantifying the number of single cells and cell‐in‐cell structures. Cells wrapped at least halfway around neighboring cells were considered to be undergoing entosis. Cell pairs participating in entosis (engulfing and invading cell) were counted as one.

The following Chemicals were used. EGTA (Biopure, 4725E), GdCl_3_ (Gadolinium (III) Chloride) (Sigma–Aldrich, 439770), AnCoA4 (Sigma–Aldrich, 532999), YM58483 (Cayman Chemical Company, 13246), SKF96365 (Cayman Chemical Company, 10009312), 2‐APB (Cayman Chemical Company, 17146), Heparin (Sigma–Aldrich, H3149), Xestospongin C (Calbiochem, 682160), saponin (Sigma–Aldrich, S7900), Forchlorfenuron (Acros, 45629), ML‐9 (Cayman Chemical Company, 10010236), Peptide‐18 (Cayman Chemical Company, 19181), and Thapsigargin (Cayman Chemical Company, 10 522).

### Live Cell Imaging of Cell‐in‐Cell Structures in Soft Agar

To track entosis, cells (2 × 10^5^ cells per mL) were embedded in growth media containing 0.3% low melting agarose and plated on micro‐insert 4 wells in polymer coverslip bottom dishes (ibidi, 80406) coated with polyHEMA. The dishes were centrifuged at 1300 rpm for 1 min and then incubated at 37 °C for 20–30 min to allow the cells to settle down. Time‐lapse microscopy was performed at 37 °C and 5% CO_2_ in live‐cell incubation chambers.

To analyze entosis time‐lapse progression, fluorescence and differential interference contrast (DIC) images were obtained every 3 s to 5 min for the indicated time courses on a LSM780 (Zeiss) confocal microscope with Zeiss Plan‐Apochromat 63×/1.4 Oil objective lens. mCherry and eGFP (GCaMP6s) were simultaneously excited at 594 and 488 nm, respectively. Fluorescence emission was collected at 615–840 nm (mCherry) and 510–570 nm (eGFP). Zen software (Zeiss) was used for image acquisition. Images were analyzed using Zen or ImageJ software.

Graphs showing low pass filtered normalized GCaMP6s intensity were generated. Raw signals were converted into values relative to the baseline (Δ*F*/*F*, where F was the baseline level). Entotic cell pairs consisting of engulfing and invading cells were counted as one. Ca^2+^ levels 3–5× above the standard deviation were recorded as Ca^2+^ signals. The time point at which a complete CIC structure was created was set to 0. The 60–0 min period, before complete cell internalized, was defined as “engulfment”. The period from 0 to 60 min after generating the CIC structures, was defined as “Cell‐in‐Cell”.

### Immunofluorescence Assay

Monolayer cells were trypsinized to a single‐cell suspension and grown on polyHEMA‐coated plates. Cells were pelleted 2.5 h later and seeded on poly‐ornithine (Sigma–Aldrich, P3655)‐coated cover glass for 10 min. After 10 min, the cells were fixed with 4% paraformaldehyde for 10 min, permeabilized with 0.02–0.1% Triton X‐100 in PBS for 10 min at 25 °C, and then blocked with 3% bovine serum albumin (BSA) in PBS for 30 min. After incubating with the indicated antibody for 2–4 h at 25 °C or overnight at 4 °C, the cells were incubated with a conjugated secondary antibody at 25 °C for 1 h. For staining PM, the cells were incubated with CellBrite Red Cytoplasmic Membrane Dyes (Biotium, 30023) at 25 °C for 10 min. For staining cytosol, the cells were incubated with CellTracker Red Dyes (Invitrogen, C34552) at 25 °C for 10 min. For staining F‐actin, the cells were incubated with Alexa FluorTM 594 Phalloidin (Invitrogen, A12381) at 25 °C for 10 min. Last, the nuclei were stained with Hoechst 33342 (Molecular Probes) for 10 min. Confocal fluorescence images were acquired using LSM780 confocal microscope (NLO; Zeiss) and LSM980 (Zeiss) with a Zeiss 63× and 100× oil objective lens (NA 1.4 and 1.46, respectively).

### Lentivirus‐Mediated Stable Cell Line Construction

HEK293T cells were plated into 12‐well plates. The cells were transfected with lentiviral constructs together with packaging plasmids (VSVg, p8.2). Supernatants were collected 48 h post‐transfection. MCF7 cells were infected with 100–500 µL of lentivirus‐containing medium and incubated for 48 h.

### Generation of the Knock‐Out Cell Lines

MCF7 Orai1 KO cells were made with a CRISPR‐Cas9 system. Guide RNA sequences for human Orai1 (sense 5’‐GATCGGCCAGAGTTACTCCGAGG‐3’ and antisense 5’‐ CCTCGGAGTAACTCTGGCCGATC ‐3’) were inserted into the pRGEN vector and pRGEN‐reporter (ToolGen). MCF7 cells were transfected with pRGEN‐Orai1, pRGEN‐Cas9, and pRGEN‐report using Lipofectamine 3000 (Invitrogen) according to the manufacturer's instructions. After 2 days, transfected cells were selected with 200 µg mL^−1^ hygromycin (PhytoTechnology Laboratories, ACR0397045A) for 7 days. Cell colonies were isolated after 2–3 weeks. To check for genome editing, the region surrounding the target site of the guide RNA was amplified using PCR (forward primer: 5’‐ATGCATCCGGAGCCCGCCCCGCCCCCGAGC‐3’ and reverse primer: 5’‐CATGGCGAAGCCGGAGAGCAG‐3’). PCR products were subsequently purified via agarose gel extraction and then sequenced. Sequencing of PCR fragments from the Orai1 KO MCF7 cells revealed 1 base‐pair deletion confirming successful Orai1 KO. (KO#1: 12 base‐pair deletion, KO#2: 1 base‐pair deletion, KO#3: 2 base‐pair insertion). Protein expression was confirmed using Western blot analysis. For immunofluorescence assay, CellTracker Red (Invitrogen, C34552) was used for labeling Orai1 KO cells.

### Total RNA Extraction, cDNA Synthesis, and Reverse Transcription‐PCR

Total RNA was extracted from the isolated cells using RIboEX (GeneAll) following the manufacturer's protocol. cDNA was synthesized from 1 µg of RNA using oligo (dT) primers and First Strand cDNA Synthesis Kit (TOYOBO). PCR amplification was conducted on the C1000 Touch thermal Cycler (Bio‐Rad) using the following amplification conditions: initial denaturation at 95 °C for 3 min and then 35–45 cycles of denaturation at 95 °C for 30 s, annealing at 55 °C for 30 s, and extension at 72 °C for 1 min; an additional extension at 72 °C for 10 min, 16 °C hold. PCR products were subsequently purified via agarose gel extraction. h.STIM1 (For 5'‐AAGGCATTACTGGCGCTGAACCATGG‐3' and Rev 5'‐ACGGGAAGAATCCAAATGTGGAGAGC‐3'), h.STIM2 (For 5'‐AACGCTGAAATGCAGCTAGCTATTGC‐3' and Rev 5'‐CGTTCTCGTAAACAAGTTGTCAACTC‐3'), h.Orai2 (For 5'‐GGCCATGGTGGAGGTGCAGCTGGAG‐3' and Rev 5'‐GAGTTCAGGTTGTGGATGTTGCT‐3'), h.Orai3 (For 5'‐TGGGTCAAGTTTGTGCCCATTGG‐3' and Rev 5'‐TGCTGCAGACGCAGAGGACCG‐3'), and GAPDH (For 5'‐CTGAACGGGAAGCTCACTGGCATG‐3' and Rev 5'‐AGGTCCACCACCCTGTTGCTGTAGC‐3')

### Western Blot Analysis

Cells were scraped into ice‐cold cell lysis buffer (20 mm Tris‐HCL pH 7.4, 150 mm NaCl, 1% Triton X‐100, 0.1% SDS), and lysed for 10 min on ice. Lysates were centrifuged at 12 000 rpm at 4 °C for 10 min. For soluble protein blotting, samples were mixed with 1/4 volume of 4× reducing sample buffer (200 mm Tris‐HCl (pH6.8), 8% SDS, 0.4% bromophenol blue, 40% glycerol, and 20% *β*‐mercaptoethanol) and boiled at 95 °C for 5 min. Samples were separated using 10% polyacrylamide SDS‐PAGE and transferred onto 0.45 µm pore size PVDF membranes (Immobilon‐P, Millipore). The membrane was blocked with TBS‐T plus 7% SKIM‐milk and incubated overnight at 4 °C with primary antibodies diluted in TBS‐T plus 3% BSA. Blots were incubated with horseradish peroxidase (HRP)‐conjugated secondary antibodies and detected using enhanced chemiluminescence (Pierce). Densitometry analysis was performed using the ImageJ software (NIH).

### Immunoprecipitation

HEK293T cells were transfected with the indicated constructs for 18 h. Transfected cells were washed three times with PBS and lysed with lysis buffer (20 mm Tris‐HCl [pH 7.4], 150 mm NaCl, and 1% Triton X‐100). Lysates were centrifuged at 12 000 rpm for 10 min and the supernatant was incubated overnight at 4 °C with anti‐Flag M2 agarose beads (Sigma–Aldrich). Lysates and immunoprecipitated samples were run on SDS‐PAGE gels, probed with horseradish peroxidase (HRP)‐conjugated secondary antibodies, and detected by enhanced chemiluminescence (Thermo Fisher Scientific).

### Antibodies and Protein Detection

Orai1 (Santa Cruz Biotechnology, SC‐377281), Orai2 (Alomone, ACC‐061), Orai3 (Alomone, ACC‐065), STIM1 (Abnova, H00006786‐M01), STIM2 (Cell Signaling Technology, 4917S), p‐MLC2 (S19) (Cell Signaling Technology, 3671S), MLC (MilliporeSigma, M4401), GAPDH (proteintech, 60004‐1‐Ig), *β*‐actin (proteintech, 66009‐1‐Ig; Santa Cruz Biotechnology, SC‐47778), SEPT2 (proteintech, 60075‐1‐Ig; Novus Biologicals, NBP1‐85212), FLAG (Sigma‐Aldrich, F1804), and GFP (MBL, 598)

### Intracellular Ca^2+^ Imaging

MCF7 cells were loaded with 1 µm of Fluo‐4 AM (Invitrogen) for 30 min at 37 °C. Ca^2+^ imaging was performed in 0 or 2 mm Ca^2+^ Ringer's solution with an IX81 microscope (Olympus) equipped with an Olympus × 40 oil objective lens (NA 1.30) and with a fluorescent arc lamp (LAMDA LS), excitation filter wheel (Lambda 10–2; Sutter Instruments), stage controller (MS‐2000; ASI), and a CCD camera (C10600; Hamamatsu) at 25 °C. Images were processed with MetaMorph software (Molecular Devices) and analyzed with excel and GraphPad Prism 5. TG (Cayman Chemical Company, 10522, 1 µm) was used for inducing ER Ca^2+^ depletion.

The changes in intracellular Ca^2+^ were determined as mean variation between the Fluo‐4 fluorescence intensities obtained during the stimulus (F) and the resting state (F0), as follows: F–F0.

### Statistical Analysis

All generated data were recorded in Excel and analyzed using GraphPad Prism 5 software. Results are presented as means ± SEM. The student's *t*‐test was used to determine pairwise statistical significance. ***p < 0.001; **p < 0.01; and *p < 0.05.

## Conflict of Interest

The authors declare no conflict of interest.

## Author Contributions

Wrote the manuscript, conceived the study, and designed and interpreted experiments: A.R.L. and C.Y.P. Performed all experiments: A.R.L.

## Supporting information

Supporting Information

Supplemental Movie 1

Supplemental Movie 2

Supplemental Movie 3

Supplemental Movie 4

Supplemental Movie 5

Supplemental Movie 6

## Data Availability

The data that support the findings of this study are available from the corresponding author upon reasonable request.

## References

[1] M. Overholtzer , A. A. Mailleux , G. Mouneimne , G. Normand , S. J. Schnitt , R. W. King , E. S. Cibas , J. S. Brugge , Cell 2007, 131, 966. 18045538 10.1016/j.cell.2007.10.040

[2] J. Durgan , Y.‐Y.u Tseng , J. C. Hamann , M.‐C. Domart , L. Collinson , A. Hall , M. Overholtzer , O. Florey , Elife 2017, 6, e27134. 28693721 10.7554/eLife.27134 PMC5505699

[3] J. C. Hamann , A. Surcel , R. Chen , C. Teragawa , J. G. Albeck , D. N. Robinson , M. Overholtzer , Cell Rep. 2017, 20, 201. 28683313 10.1016/j.celrep.2017.06.037 PMC5559205

[4] M. Krajcovic , N. B. Johnson , Q. Sun , G. Normand , N. Hoover , E. Yao , A. L. Richardson , R. W. King , E. S. Cibas , S. J. Schnitt , J. S. Brugge , M. Overholtzer , Nat. Cell Biol. 2011, 13, 324. 21336303 10.1038/ncb2174 PMC3576821

[5] H. L. Mackay , D. Moore , C. Hall , N. J. Birkbak , M. Jamal‐Hanjani , S. A. Karim , V. M. Phatak , L. Piñon , J. P. Morton , C. Swanton , J. Le Quesne , P. A. J. Muller , Nat. Commun. 2018, 9, 3070. 30076358 10.1038/s41467-018-05368-1 PMC6076230

[6] Y. Li , X. Sun , S. K. Dey , Cell Rep. 2015, 11, 358. 25865893 10.1016/j.celrep.2015.03.035 PMC5089169

[7] Y. Lee , J. C. Hamann , M. Pellegrino , J. Durgan , M.‐C. Domart , L. M. Collinson , C. M. Haynes , O. Florey , M. Overholtzer , Cell Rep. 2019, 26, 3212. 30893595 10.1016/j.celrep.2019.02.073 PMC6475604

[8] S. Krishna , M. Overholtzer , Cell. Mol. Life Sci. 2016, 73, 2379. 27048820 10.1007/s00018-016-2207-0 PMC4889469

[9] Q. Sun , E. S. Cibas , H. Huang , L. Hodgson , M. Overholtzer , Cell Res. 2014, 24, 1288. 25342558 10.1038/cr.2014.137 PMC4220160

[10] W. Ren , W. Zhao , L. Cao , J. Huang , Front. Cell Dev. Biol. 2020, 8, 634849. 33634110 10.3389/fcell.2020.634849 PMC7900405

[11] M. Wang , Z. Niu , H. Qin , B. Ruan , Y. Zheng , X. Ning , S. Gu , L. Gao , Z. Chen , X. Wang , H. Huang , L.i Ma , Q. Sun , Cell Rep. 2020, 32, 108071. 32846129 10.1016/j.celrep.2020.108071

[12] O. Florey , S. E. Kim , C. P. Sandoval , C. M. Haynes , M. Overholtzer , Nat. Cell Biol. 2011, 13, 1335. 22002674 10.1038/ncb2363 PMC3223412

[13] Q. Sun , T. Luo , Y. Ren , O. Florey , S. Shirasawa , T. Sasazuki , D. N. Robinson , M. Overholtzer , Cell Res. 2014, 24, 1299. 25342560 10.1038/cr.2014.138 PMC4220161

[14] V. Purvanov , M. Holst , J. Khan , C. Baarlink , R. Grosse , Elife 2014, 3, e02786. 24950964 10.7554/eLife.02786 PMC4091095

[15] M. J. Berridge , M. D. Bootman , H. L. Roderick , Nat. Rev. Mol. Cell Biol. 2003, 4, 517. 12838335 10.1038/nrm1155

[16] D. E. Clapham , Cell 2007, 131, 1047. 18083096 10.1016/j.cell.2007.11.028

[17] Y.‐F. Chen , Y.‐T. Chen , W.‐T. Chiu , M.‐R.u Shen , J. Biomed. Sci. 2013, 20, 23. 23594099 10.1186/1423-0127-20-23 PMC3639169

[18] R. Bagur , G. Hajnóczky , Mol. Cell 2017, 66, 780. 28622523 10.1016/j.molcel.2017.05.028 PMC5657234

[19] M. Prakriya , S. Feske , Y. Gwack , S. Srikanth , A. Rao , P. G. Hogan , Nature 2006, 443, 230. 16921383 10.1038/nature05122

[20] S. Feske , Y. Gwack , M. Prakriya , S. Srikanth , S.‐H. Puppel , B. Tanasa , P. G. Hogan , R. S. Lewis , M. Daly , A. Rao , Nature 2006, 441, 179. 16582901 10.1038/nature04702

[21] S. L. Zhang , Y. Yu , J. Roos , J. A. Kozak , T. J. Deerinck , M. H. Ellisman , K. A. Stauderman , M. D. Cahalan , Nature 2005, 437, 902. 16208375 10.1038/nature04147 PMC1618826

[22] S. Yang , J. J. Zhang , X.‐Y. Huang , Cancer Cell 2009, 15, 124. 19185847 10.1016/j.ccr.2008.12.019

[23] M. Prakriya , R. S. Lewis , Physiol. Rev. 2015, 95, 1383. 26400989 10.1152/physrev.00020.2014 PMC4600950

[24] P. G. Hogan , Cell Calcium 2015, 58, 357. 26215475 10.1016/j.ceca.2015.07.001 PMC4564343

[25] R. M. L. La Rovere , G. Roest , G. Bultynck , J. B. Parys , Cell Calcium 2016, 60, 74. 27157108 10.1016/j.ceca.2016.04.005

[26] S. Mostowy , P. Cossart , Nat. Rev. Mol. Cell Biol. 2012, 13, 183. 22314400 10.1038/nrm3284

[27] A. A. Bridges , M. S. Jentzsch , P. W. Oakes , P. Occhipinti , A. S. Gladfelter , J. Cell Biol. 2016, 213, 23. 27044896 10.1083/jcb.201512029 PMC4828694

[28] D. Lobato‐Márquez , S. Mostowy , J. Cell Biol. 2016, 213, 5. 27044893 10.1083/jcb.201603063 PMC4828695

[29] S. Sharma , A. Quintana , G. M. Findlay , M. Mettlen , B. Baust , M. Jain , R. Nilsson , A. Rao , P. G. Hogan , Nature 2013, 499, 238. 23792561 10.1038/nature12229 PMC3846693

[30] M. I. Lioudyno , J. A. Kozak , A. Penna , O. Safrina , S. L. Zhang , D. Sen , J. Roos , K. A. Stauderman , M. D. Cahalan , Proc. Natl. Acad. Sci. U. S. A. 2008, 105, 2011. 18250319 10.1073/pnas.0706122105 PMC2538873

[31] K. Aoki , S. Harada , K. Kawaji , K. Matsuzawa , S. Uchida , J. Ikenouchi , Nat. Commun. 2021, 12, 480. 33473127 10.1038/s41467-020-20826-5 PMC7817837

[32] F.‐C. Tsai , A. Seki , H. W. Yang , A. Hayer , S. Carrasco , S. Malmersjö , T. Meyer , Nat. Cell Biol. 2014, 16, 133. 24463606 10.1038/ncb2906 PMC3953390

[33] A. M. Lopez‐Guerrero , N. Espinosa‐Bermejo , I. Sanchez‐Lopez , T. Macartney , C. Pascual‐Caro , Y. Orantos‐Aguilera , L. Rodriguez‐Ruiz , A. B. Perez‐Oliva , V. Mulero , E. Pozo‐Guisado , F. J. Martin‐Romero , Sci. Rep. 2020, 10, 6580. 32313105 10.1038/s41598-020-63353-5 PMC7171199

[34] Y.‐T. Chen , Y.‐F. Chen , W.‐T. Chiu , Y.‐K. Wang , H.‐C. Chang , M.‐R.u Shen , J. Cell Sci. 2013, 126, 1260. 23378028 10.1242/jcs.121129

[35] T.‐W. Chen , T. J. Wardill , Y.i Sun , S. R. Pulver , S. L. Renninger , A. Baohan , E. R. Schreiter , R. A. Kerr , M. B. Orger , V. Jayaraman , L. L. Looger , K. Svoboda , D. S. Kim , Nature 2013, 499, 295. 23868258 10.1038/nature12354 PMC3777791

[36] A. M. Sadaghiani , S. M. Lee , J. I. Odegaard , D. B. Leveson‐Gower , O. M. Mcpherson , P. Novick , M. R. Kim , A. N. Koehler , R. Negrin , R. E. Dolmetsch , C. Y. Park , Chem. Biol. 2014, 21, 1278. 25308275 10.1016/j.chembiol.2014.08.016

[37] J. J. Lopez , L. Albarran , L. J. Gómez , T. Smani , G. M. Salido , J. A. Rosado , Biochim. Biophys. Acta 2016, 1863, 2037. 27130253 10.1016/j.bbamcr.2016.04.024

[38] Z. B. Katz , C. Zhang , A. Quintana , B. F. Lillemeier , P. G. Hogan , Sci. Rep. 2019, 9, 10839. 31346209 10.1038/s41598-019-46862-w PMC6658532

[39] Q. Hu , W. J. Nelson , E. T. Spiliotis , J. Biol. Chem. 2008, 283, 29563. 18713753 10.1074/jbc.M804962200 PMC2570864

[40] V. K. Sidhaye , E. Chau , P. N. Breysse , L. S. King , Am. J. Respir. Cell Mol. Biol. 2011, 45, 120. 20870893 10.1165/rcmb.2010-0235OC PMC3145065

[41] N. F. Valadares , H. D’ Muniz Pereira , A. P. Ulian Araujo , R. C. Garratt , Biophys. Rev. 2017, 9, 481. 28905266 10.1007/s12551-017-0320-4 PMC5662055

[42] R. K. Motiani , I. F. Abdullaev , M. Trebak , J. Biol. Chem. 2010, 285, 19173. 20395295 10.1074/jbc.M110.102582 PMC2885196

[43] H. L. Ong , K. T. Cheng , X. Liu , B. C. Bandyopadhyay , B. C. Paria , J. Soboloff , B. Pani , Y. Gwack , S. Srikanth , B. B. Singh , D. Gill , I. S. Ambudkar , J. Biol. Chem. 2007, 282, 9105. 17224452 10.1074/jbc.M608942200 PMC3309402

[44] Y. Liao , C. Erxleben , E. Yildirim , J. Abramowitz , D. L. Armstrong , L. Birnbaumer , Proc. Natl. Acad. Sci. U. S. A. 2007, 104, 4682. 17360584 10.1073/pnas.0611692104 PMC1838661

[45] Y. Liu , X. Zheng , G. A. Mueller , M. Sobhany , E. F. Derose , Y. Zhang , R. E. London , L. Birnbaumer , J. Biol. Chem. 2012, 287, 43030. 23109337 10.1074/jbc.M112.380964 PMC3522297

[46] A. Jha , M. Ahuja , J. Maléth , C. M. Moreno , J. P. Yuan , M. S. Kim , S. Muallem , J. Cell Biol. 2013, 202, 71. 23816623 10.1083/jcb.201301148 PMC3704993

[47] L. Albarran , J. J. Lopez , I. Jardin , J. Sanchez‐Collado , A. Berna‐Erro , T. Smani , P. J. Camello , G. M. Salido , J. A. Rosado , Cell. Physiol. Biochem. 2018, 51, 1164. 30481768 10.1159/000495494

[48] A. M. Lopez‐Guerrero , P. Tomas‐Martin , C. Pascual‐Caro , T. Macartney , A. Rojas‐Fernandez , G. Ball , D. R. Alessi , E. Pozo‐Guisado , F. J. Martin‐Romero , Sci. Rep. 2017, 7, 383. 28341841 10.1038/s41598-017-00331-4 PMC5428229

[49] S. Pece , J. S. Gutkind , J. Biol. Chem. 2000, 275, 41227. 10969083 10.1074/jbc.M006578200

[50] R. R. Rayavarapu , B. Heiden , N. Pagani , M. M. Shaw , S. Shuff , S. Zhang , Z. T. Schafer , J. Biol. Chem. 2015, 290, 8722. 25681438 10.1074/jbc.M114.612754 PMC4423663

[51] A. Sharma , R. C. Elble , Biomedicines 2020, 8, 169. 32575848 10.3390/biomedicines8060169 PMC7345168

[52] M. Vandenberghe , M. Raphaël , V. Lehen'kyi , D. Gordienko , R. Hastie , T. Oddos , A. Rao , P. G. Hogan , R. Skryma , N. Prevarskaya , Proc. Natl. Acad. Sci. U. S. A. 2013, 110, E4839. 24277812 10.1073/pnas.1310394110 PMC3864283

[53] S. M. Emrich , R. E. Yoast , P. Xin , V. Arige , L. E. Wagner , N. Hempel , D. L. Gill , J. Sneyd , D. I. Yule , M. Trebak , Cell Rep. 2021, 34, 108760. 33657364 10.1016/j.celrep.2021.108760 PMC7968378

[54] J. Sun , F. Lu , H. He , J. Shen , J. Messina , R. Mathew , D. Wang , A. A. Sarnaik , W.‐C. Chang , M. Kim , H. Cheng , S. Yang , J. Cell Biol. 2014, 207, 535. 25404747 10.1083/jcb.201407082 PMC4242838

[55] R. E. Yoast , S. M. Emrich , X. Zhang , P. Xin , M. T. Johnson , A. J. Fike , V. Walter , N. Hempel , D. I. Yule , J. Sneyd , D. L. Gill , M. Trebak , Nat. Commun. 2020, 11, 2444. 32415068 10.1038/s41467-020-16232-6 PMC7229178

[56] M. J. Berridge , Physiol. Rev. 2016, 96, 1261. 27512009 10.1152/physrev.00006.2016

[57] A. Politi , L. D. Gaspers , A. P. Thomas , T. Höfer , Biophys. J. 2006, 90, 3120. 16500959 10.1529/biophysj.105.072249 PMC1432125

[58] C. Fewtrell , Annu. Rev. Physiol. 1993, 55, 427. 8385436 10.1146/annurev.ph.55.030193.002235

