# Supplementary material for: Orai1 is an Entotic Ca2+ Channel for Non‐Apoptotic Cell Death, Entosis in Cancer Development
Source: Adv Sci (Weinh). 2023 Mar 24;10(14):2205913. doi: 10.1002/advs.202205913 (PMC10190626; doi:10.1002/advs.202205913)
Supplement: Supplementary file 1 — Supporting Information [file ADVS-10-2205913-s007.pdf]

ADVANCED SCIENCE
Open Access

Supporting Information

for *Adv. Sci.*, DOI 10.1002/advs.202205913

Orai1 is an Entotic Ca^{2+} Channel for Non-Apoptotic Cell Death, Entosis in Cancer Development

*Ah Reum Lee and Chan Young Park**

Supporting Information

Orai1 is an entotic Ca^{2+} channel for non-apoptotic cell death, entosis in cancer development.

Ah Reum Lee[1] and Chan Young Park[1] *

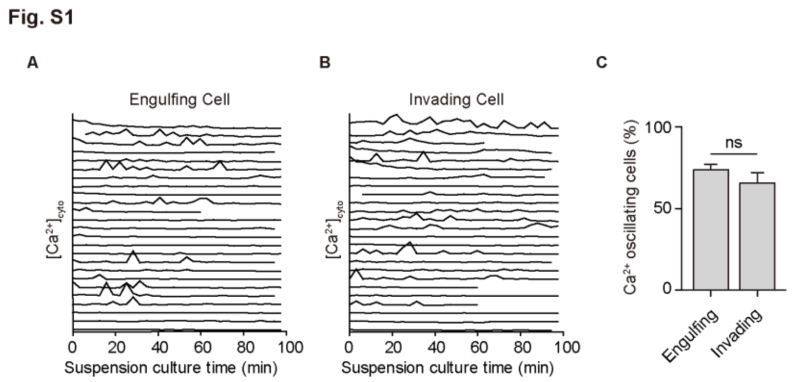

Figure S1. Entotic Ca^{2+} oscillations in engulfing and invading cells.
(**A** and **B**) Graphs of normalized GCaMP6s ratio in engulfing (A) and invading (B) cells. (n > 22) (**C**) Quantification of Ca^{2+} oscillating cells from (A and B). Significance was determined using unpaired two-tailed *t*-test. ns, not significant.

Fig. S2

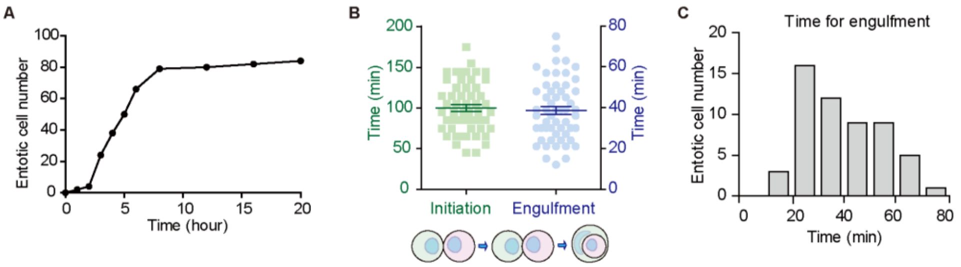

Figure S2. Time kinetics of the initiation of internalization and engulfment.
(**A**) Quantification of entotic cells using time-lapse imaging conducted over 20 h (n = 84). (**B** and **C**) Time to entosis initiation and engulfment. Engulfment was initiated after 1 - 2 h of suspension (n = 54) (B). Complete engulfment takes 30 - 60 min after the initiation (n = 55) (B and C).

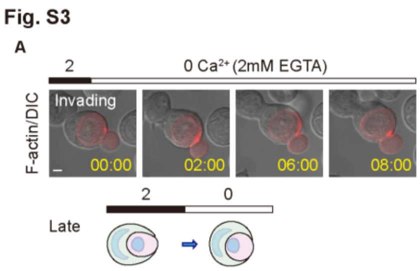

Figure S3. Entotic cells in Ca^{2+} withdrawal condition.
(**A**) Time-lapse images of entotic cells under Ca^{2+} withdrawal (2 -> 0 mM). Cherry-Lifeact-labeled cell morphology. Scale bar = 5 µm.

Fig. S4

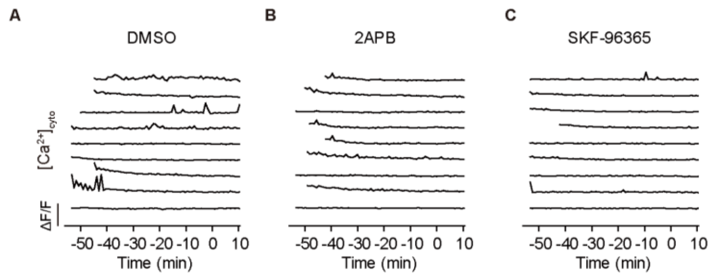

Figure S4. Ca^{2+} oscillations in SOCE inhibitors.

(**A** to **C**) Graphs of normalized GCaMP6s ratio in SOCE inhibitors. DMSO (A, n = 28), 50 µM 2-APB (B, n = 19) and 10 µM SKF96365 (C, n = 24) were added on the culture media. The time-point marking the complete cell-in-cell structure was set to 0. Images were taken at 30 s intervals.

Fig. S5

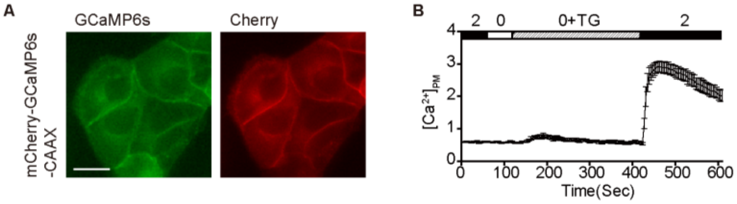

Figure S5. Ca^{2+} signals in plasma membrane.

(**A**) Fluorescence images of mCherry-GCaMP6s-CAAX. Scale bar = 20 µm. (**B**) Thapsigargin (TG)-induced Ca^{2+} influx in MCF-7 cells monitored using GCaMP6s-CAAX/Cherry Fluorescence ratio.

Fig. S6

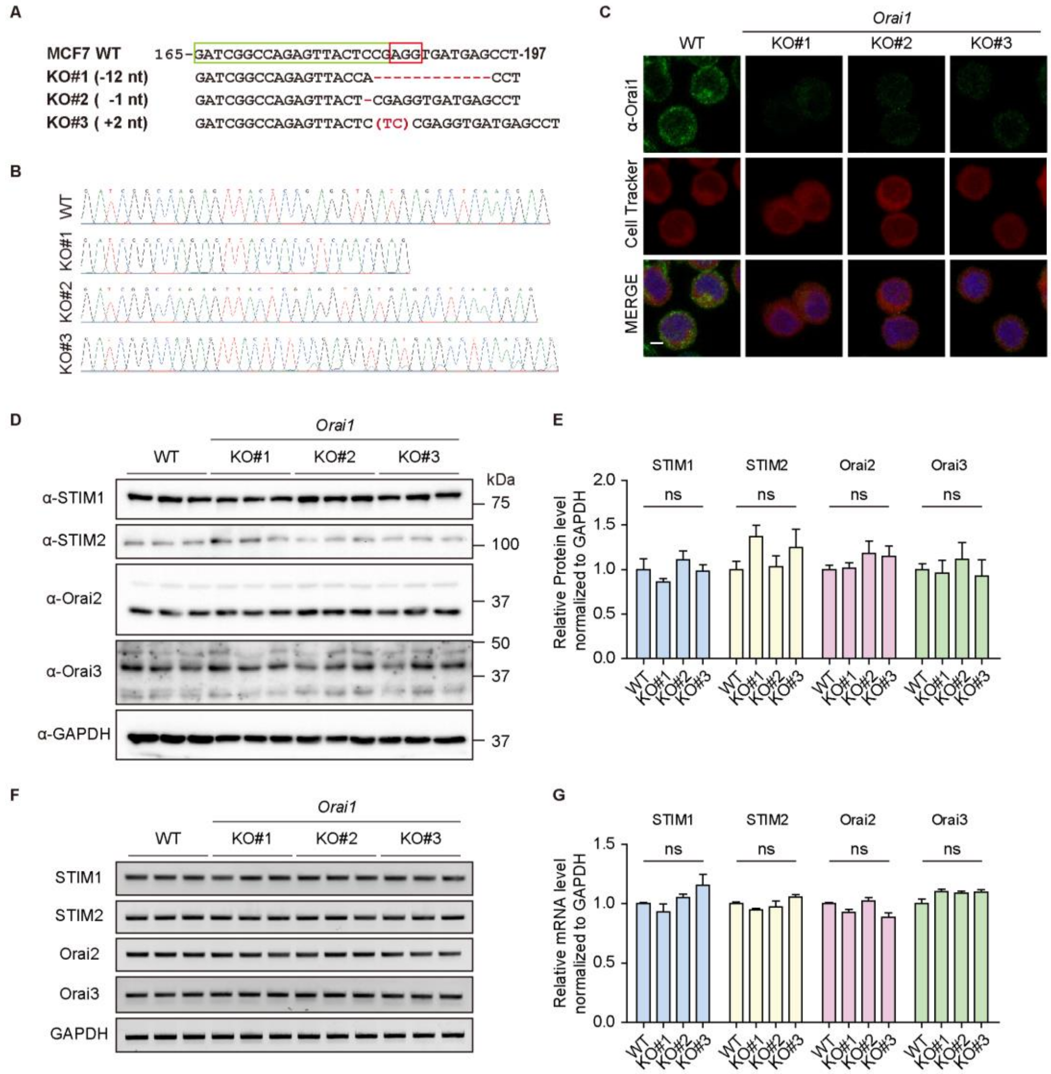

Figure S6. Genetic deletion of *Orai1* using CRISPR-Cas9 system.

(**A** and **B**) Sequences of *Orai1* KO MCF7 cells. Alignment of human *Orai1* sequences from *Orai1* KO cell lines used in this study. (**C**) Immunofluorescence images of endogenous Orai1 (green). Non-labeled Orai1 WT cells were mixed with Cell-Tracker Red labeled *Orai1* KO cell lines. (**D** and **F**) Protein (D) and mRNA (F) expression levels of other SOCE components: STIM1, STIM2, Orai2, and Orai3 in *Orai1* KO cell lines. GAPDH was used as endogenous control. (**E** and **G**) Relative protein (E, from D) and mRNA (G, from F) expression level of SOCE components normalized with GAPDH. Statistical analysis was performed using one-way ANOVA followed by Dunnett’s test. ns, not significant.

Fig. S7

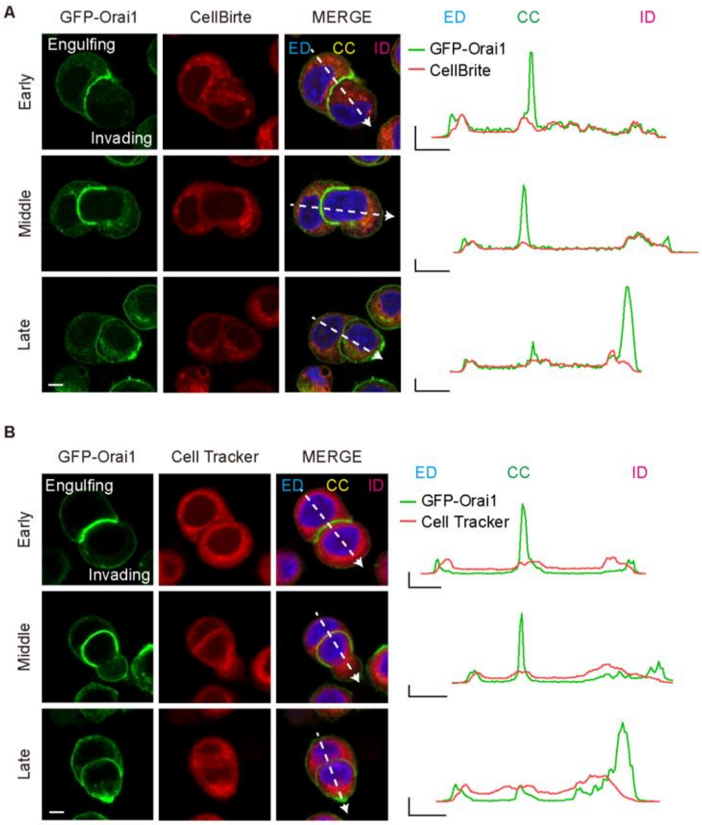

Figure S7. The polarized distribution of Orai1 during entosis.

(**A** and **B**) Fluorescence images of GFP-Orai1 expressing MCF7 cells stained with CellBrite PM marker (A, red, PM) or CellTracker (B, red, cytosol). Line scan analysis of relative GFP-Orai1 (green) and PM or cytosol marker (red) signals along the white arrow in the merge image. X axis: 5 µm, Y axis: 1 (A.U.). Scale bar = 5 µm. ED, Engulfing cell distal region; CC, cell-cell contact site; ID, Invading cell distal region.

Fig. S8

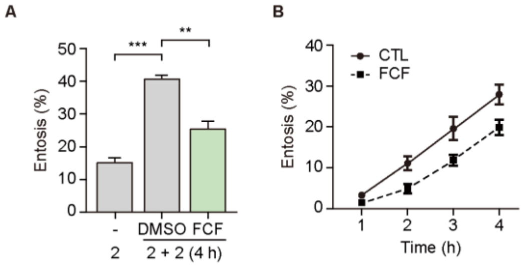

Figure S8. SEPTIN inhibitor suppresses entosis.

(**A**) Quantification of internalized cells suspended in FCF. Cells were pre-suspended for 2 h and then mixed with FCF and cultured for an additional 2 h. Data represent mean ± SEM of triplicate experiments (n > 200 in each experiment). Significance was determined using unpaired two-tailed *t*-test. ***p < 0.001, **p < 0.01 (**B**) Time-dependent quantification of entotic cells with or without FCF. MCF7 cells were cultured in suspension for 4 h at 1 h intervals. Data represent mean ± SEM of triplicate experiments (n > 300 in each experiment).

Fig. S9

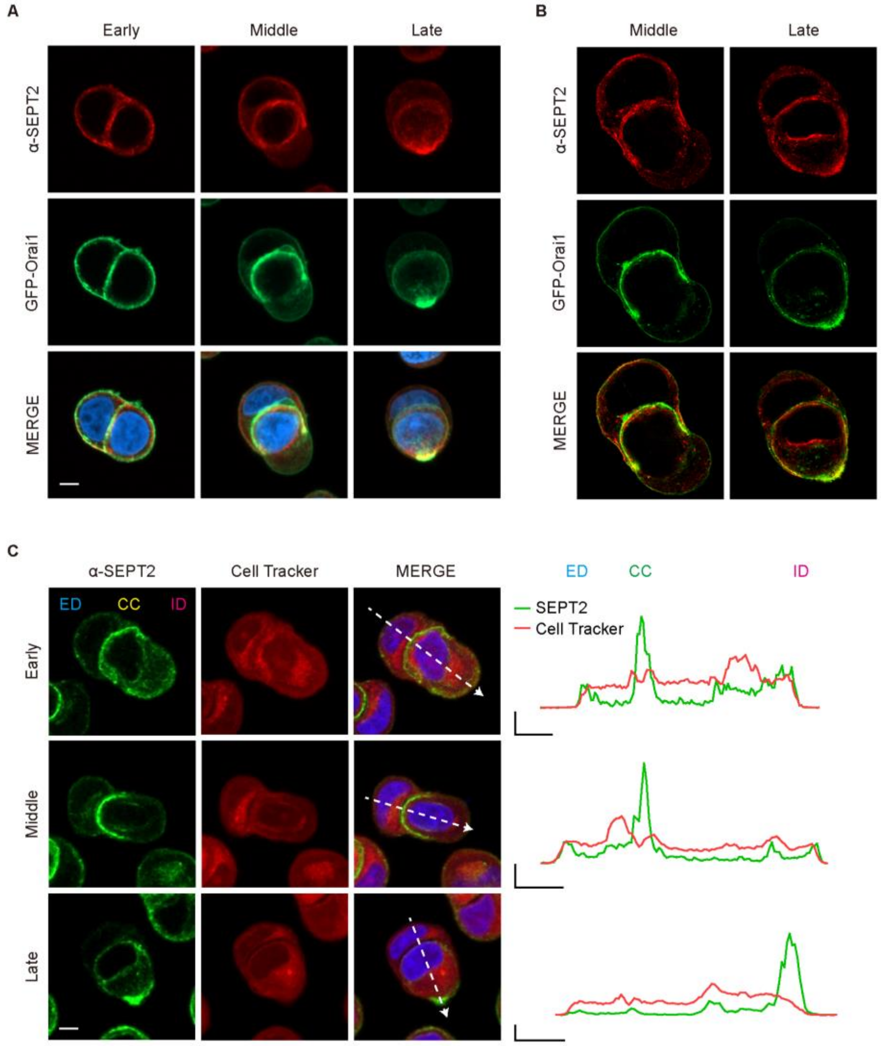

Figure S9. SEPT2 co-localizes with Orai1.

(**A**) 3D projection of immunofluorescence images showing endogenous SEPT2 (red) and GFP-Orai1 (green) (**B**) Super-resolution microscopy of the localization of SEPT2 (red) and GFP-Orai1 (green) during entosis. (**C**) Immuno-fluorescent images of suspended cells taken after 2.5 h of culturing show endogenous SEPT2 (green) and CellTracker (red, cytosol marker). Line graphs show SEPT2 (green) and cytosol marker (red) intensities for the indicated line scans. X axis: 5 µm, Y axis: 1 (A.U.). Scale bar = 5 µm. ED, Engulfing cell distal region; CC, cell-cell contact site; ID, Invading cell distal region.

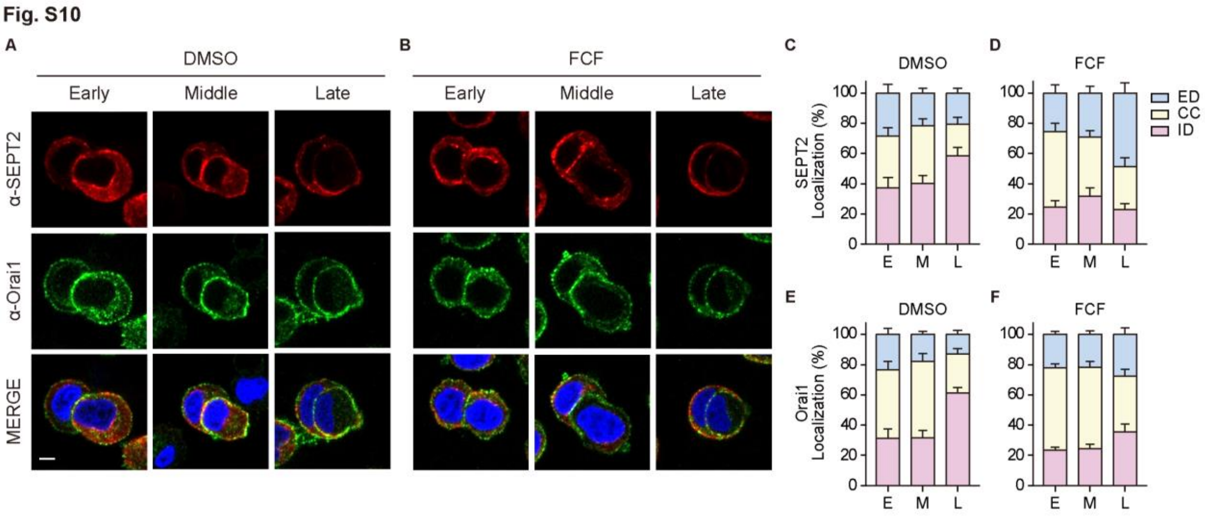

Figure S10. SEPTIN inhibitor suppresses Orai1 organization.

(**A** and **B**) Immunofluorescence images showing endogenous SEPT2 (red) and endogenous Orai1 (green) in MCF7 cells cultured for 2.5 h in DMSO (A) or FCF (B, 50 μM). Scale bar = 5 μm. (**C** to **F**) Bar graphs showing the distribution of SEPT2 (C, DMSO; D, FCF) and Orai1 (E, DMSO; F**,** FCF). n = 34 in the DMSO group; n = 29 in the FCF group. ED, Engulfing cell distal region; CC, cell-cell contact site; ID, Invading cell distal region. E, Early; M, Middle; L, Late.

Fig. S11

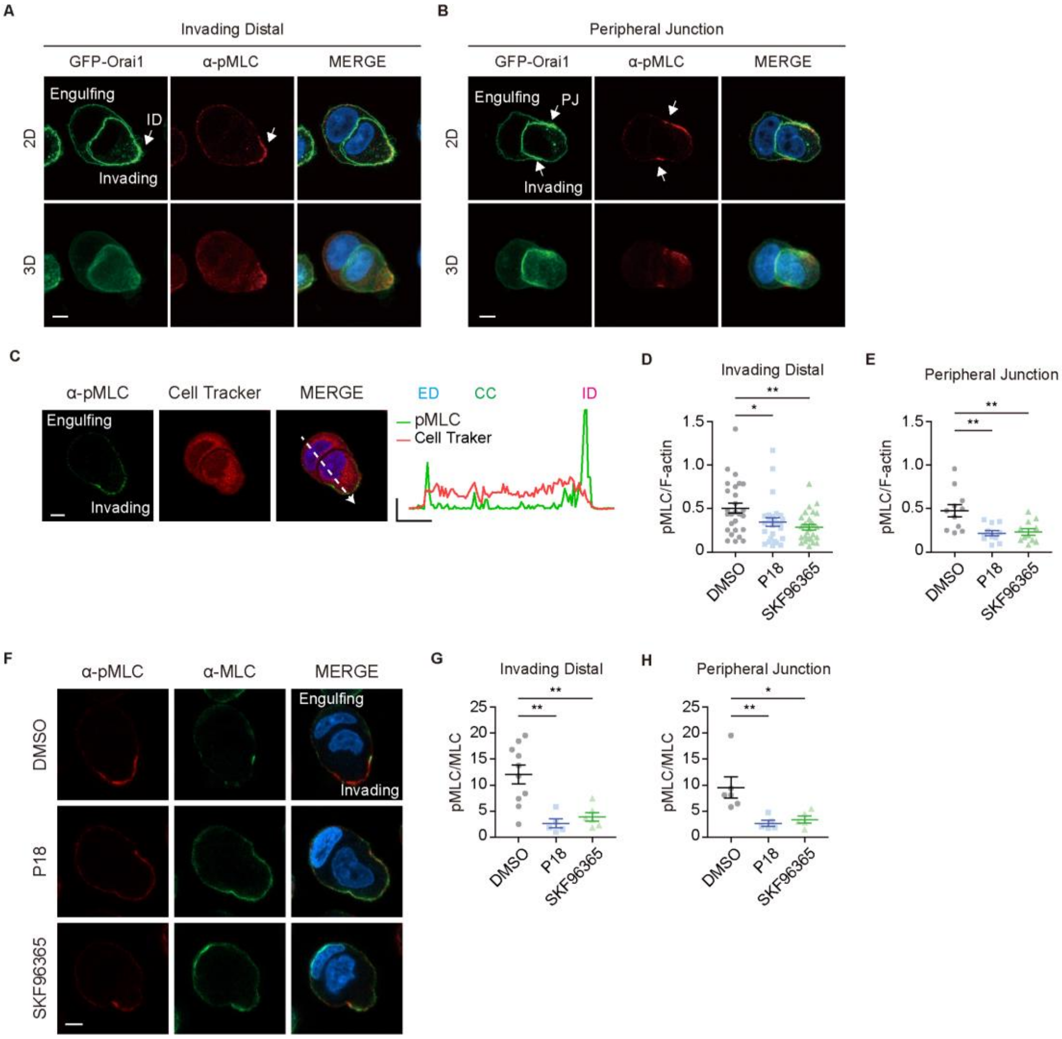

Figure S11. MLC phosphorylation is induced by Orai1 at invading cell distal region and peripheral junction.

(**A** and **B**) Immunofluorescence images showing endogenous pMLC (red) in GFP-Orai1 expressing MCF7 cells cultured in suspension for 2.5 h. pMLC is enriched at ID (A) and PJ (B). (**C**) Immuno-fluorescent images of suspended cells taken after 2.5 h of culturing show endogenous pMLC (green) and CellTracker (red, cytosol marker). Line graphs show relative pMLC (green) and cytosol marker (red) intensities for the indicated line scans. X axis: 5 µm, Y axis: 5 (A.U.). (**D** and **E**) Quantification of pMLC/F-actin intensity ratio at ID (D, from Figure6E**,** n=27 in each group) and PJ (E, from Figure6F, n=11 in each group). (**F**) Immuno-fluorescence images of suspended cells after 2.5 h of culturing show endogenous pMLC (red) and MLC (green) in MCF7 cells. (**G** and **H**) Quantification of pMLC/MLC intensity ratio at ID (G, n=10, 5, 5) and PJ (H, n=6, 5, 5). Scale bar = 5 µm. ED, Engulfing cell distal region; CC, cell-cell contact site; ID, Invading cell distal region; PJ, peripheral junction. Significance was determined using unpaired two-tailed *t*-test. ***$p < 0.001$, **$p < 0.01$, *$p < 0.05$.

Movie S1
Cytosolic Ca^{2+} oscillations in Cherry-GCaMP6s expressing MCF7 cells. Scale bar = 5 µm.

Movie S2
Plasma membrane Ca^{2+} oscillations in Cherry-GCaMP6s-CAAX expressing engulfing MCF7 cell. Scale bar = 5 µm.

Movie S3
Plasma membrane Ca^{2+} oscillations in Cherry-GCaMP6s-CAAX expressing invading MCF7 cell. Scale bar = 5 µm.

Movie S4
Plasma membrane Ca^{2+} oscillations in Cherry-GCaMP6s-CAAX expressing *Orai1* WT MCF7 cells. Scale bar = 20 µm.

Movie S5
Plasma membrane Ca^{2+} oscillations in Cherry-GCaMP6s-CAAX expressing *Orai1* KO MCF7 cells. Scale bar = 20 µm.

Movie S6
Orai1 localization in GFP-Orai1 expressing MCF7 cells. Scale bar = 5 µm.
